# Long-term efficacy, tolerability and overall survival in patients with platinum-sensitive, recurrent high-grade serous ovarian cancer treated with maintenance olaparib capsules following response to chemotherapy

**DOI:** 10.1038/s41416-018-0271-y

**Published:** 2018-10-24

**Authors:** Michael Friedlander, Ursula Matulonis, Charlie Gourley, Andreas du Bois, Ignace Vergote, Gordon Rustin, Clare Scott, Werner Meier, Ronnie Shapira-Frommer, Tamar Safra, Daniela Matei, Vadim Shirinkin, Frédéric Selle, Anitra Fielding, Elizabeth S. Lowe, Emma L. McMurtry, Stuart Spencer, Philip Rowe, Helen Mann, David Parry, Jonathan Ledermann

**Affiliations:** 1 University of New South Wales Clinical School, Prince of Wales Hospital, Randwick, NSW Australia; 2 Dana-Farber Cancer Institute, Boston, MA USA; 3 Cancer Research UK Edinburgh Centre, Western General Hospital, Edinburgh, UK; 4 Kliniken Essen Mitte, Essen, Germany; 5 University of Leuven, Leuven Cancer Institute, Leuven, Belgium; 6 Mount Vernon Hospital, Northwood, UK; 7 Royal Melbourne Hospital, Parkville, VIC Australia; 8 University of Düsseldorf, Düsseldorf, Germany; 9 Chaim Sheba Medical Center, Tel Hashomer, Israel; 10 Tel Aviv Sourasky Medical Center, Tel Aviv, Israel; 11 Sackler School of Medicine, Tel Aviv University, Tel Aviv, Israel; 12 Northwestern University Feinberg School of Medicine, Chicago, IL USA; 13 Orenburg Regional Clinical Oncological Dispensary, Orenburg, Russia; 14 Groupe Hospitalier Diaconesses Croix Saint-Simon, Paris, France; 15 GINECO Group, Paris, France; 16 AstraZeneca, Cambridge, UK; 17 AstraZeneca, Gaithersburg, MD USA; 18 University College London Cancer Institute, London, UK; 19 Present Address: AstraZeneca, Gaithersburg, MD USA

## Abstract

**Background:**

In Study 19, maintenance monotherapy with olaparib significantly prolonged progression-free survival vs placebo in patients with platinum-sensitive, recurrent high-grade serous ovarian cancer.

**Methods:**

Study 19 was a randomised, placebo-controlled, Phase II trial enrolling 265 patients who had received at least two platinum-based chemotherapy regimens and were in complete or partial response to their most recent regimen. Patients were randomised to olaparib (capsules; 400 mg bid) or placebo. We present long-term safety and final mature overall survival (OS; 79% maturity) data, from the last data cut-off (9 May 2016).

**Results:**

Thirty-two patients (24%) received maintenance olaparib for over 2 years; 15 (11%) did so for over 6 years. No new tolerability signals were identified with long-term treatment and adverse events were generally low grade. The incidence of discontinuations due to adverse events was low (6%). An apparent OS advantage was observed with olaparib vs placebo (hazard ratio 0.73, 95% confidence interval 0.55‒0.95, *P* *=* 0.02138) irrespective of *BRCA1/2* mutation status, although the predefined threshold for statistical significance was not met.

**Conclusions:**

Study 19 showed a favourable final OS result irrespective of *BRCA1/2* mutation status and unprecedented long-term benefit with maintenance olaparib for a subset of platinum-sensitive, recurrent ovarian cancer patients.

## Introduction

Ovarian cancer is the most common cause of gynaecological cancer-related deaths and fifth leading cause of death from cancer in women.^1,2^ Median overall survival (OS) for patients with platinum-sensitive, recurrent ovarian cancer (defined as relapse ≥ 6 months after platinum-based chemotherapy) is 2.5–3 years, and patients typically receive a median of four lines of chemotherapy after progression.^3,4^ Progression-free survival (PFS) for these patients ranges from 8 to 13 months from the start of second-line chemotherapy.^3^ In patients who respond to further platinum-based chemotherapy, the median PFS from the end of treatment is consistently 5–6 months^5–7^ and most patients are offered the next line of palliative chemotherapy. Cumulative toxicities of chemotherapy and the emergence of drug resistance limit delivery and potential benefit of further treatment. Furthermore, the duration of benefit associated with ‘salvage’ chemotherapy for progression decreases with each subsequent line of treatment.^8,9^ There remains an unmet need for effective and well-tolerated long-term maintenance treatment options for patients with recurrent ovarian cancer to maintain quality of life and delay the need for further chemotherapy, particularly after response.

Olaparib (Lynparza™), a poly(adenosine diphosphate–ribose) polymerase (PARP) inhibitor, is approved (tablet formulation) for treatment in the maintenance setting for patients with platinum-sensitive relapsed ovarian cancer, irrespective of *BRCA1/2* mutation (*BRCA*m) status.^10,11^

We have previously reported data from Study 19 (NCT00753545), a Phase II trial that assessed the efficacy and safety of olaparib maintenance monotherapy in platinum-sensitive, recurrent high-grade serous ovarian cancer patients, and showed a significant improvement in PFS with maintenance olaparib vs placebo (hazard ratio (HR) 0.35, 95% confidence interval (CI) 0.25–0.49; *P* *<* 0.0001).^5^ The PFS prolongation was durable, with 32 of 136 patients (24%) who received olaparib being progression-free for >2 years.^12^ A pre-planned, retrospective analysis of patients in Study 19 demonstrated that *BRCA*m patients derived the greatest clinical benefit from olaparib (HR 0.18, 95% CI 0.10–0.31; *P* *<* 0.0001); however, a PFS advantage was also seen for *BRCA* wild-type (*BRCA*wt) patients (HR 0.54, 95% CI 0.34–0.85; *P* *=* 0.0075).^13^ More recently, PFS data from the Phase III SOLO2 trial of olaparib tablets as maintenance monotherapy in patients with platinum-sensitive, relapsed ovarian cancer and a *BRCA*m confirmed a significant benefit for olaparib-treated patients compared with those who received placebo (HR 0.30, 95% CI 0.22–0.41; *P* *<* 0.0001).^6^ Although the initial analysis of OS in Study 19 (data maturity: 38%) did not detect a treatment effect, previously reported updated data from an interim analysis (data cut-off [DCO] 30 September 2015; data maturity: 77%) suggested an advantage in OS for patients receiving olaparib vs placebo (HR 0.73, 95% CI 0.55–0.96; nominal *P* *=* 0.025).^5,14^

Safety data from clinical trials in recurrent ovarian cancer patients have shown that olaparib (capsule) monotherapy is generally well tolerated, and data from SOLO2 suggest the new tablet formulation of olaparib has a similar tolerability profile.^6,14–18^ Furthermore, there was no detriment to health-related quality of life (HRQoL) while on treatment in either Study 19 or SOLO2.^19,20^ Given its clinical profile, long-term administration of olaparib as maintenance monotherapy in ovarian cancer patients is feasible and an attractive option. Here, we report the final protocol-defined OS analysis from Study 19, characterise those patients who have derived long-term benefit from olaparib and, in particular, assess the long-term safety and tolerability of olaparib treatment.

## Materials and methods

### Study design and population

Study 19 was a Phase II, randomised, double-blind, placebo-controlled trial (NCT00753545). Eligible patients were at least 18 years old, with recurrent ovarian, fallopian tube or primary peritoneal cancer with high-grade serous histology and were platinum sensitive. Patients had received two or more prior courses of platinum-based chemotherapy and were in complete or partial response to their most recent regimen (Supplementary Methods). Additional eligibility criteria have been described previously.^5^ Known *BRCA*m status was not required but was established retrospectively by germline or tumour testing for patients with appropriate samples available who provided consent (Supplementary Methods).

### Treatments

Patients were randomised 1:1 to olaparib or matching placebo, as described previously.^5^ Olaparib maintenance monotherapy (400 mg bid, capsule formulation) was administered orally and treatment continued until disease progression, if toxicities were manageable (Supplementary Methods).

### Assessments

Tumour assessments were conducted every 12 weeks until week 60, and every 24 weeks thereafter, until objective disease progression or withdrawal of consent. Response Evaluation Criteria in Solid Tumours (RECIST) data were not collected after the primary DCO (30 June 2010). The primary endpoint of the study was PFS, for which data have been reported previously.^5^ OS was a secondary endpoint and we report the final analysis for this outcome here. Patients were monitored for OS with follow-up every 12 weeks after treatment discontinuation. Exploratory analyses of time to first and second subsequent therapy or death (TFST, TSST) were also conducted. Safety and tolerability were assessed throughout the study by recording adverse events (AEs; graded using CTCAE v3.0), physical examination results, vital signs and laboratory findings.

### Statistical analyses

Study 19 was powered to ensure a sufficient number of PFS events in the overall study population; it was not formally powered to assess differences in OS, either between active treatment and placebo or within different patient groups. Previous OS analyses are listed in the Supplementary Methods. The final OS analysis for Study 19, reported here, (79% data maturity; DCO: 9 May 2016; two-sided *α* = 0.95%) was protocol defined and the analysis set for OS included all patients randomised to treatment. The results should be regarded as descriptive and *P*-values as nominal. OS, TFST and TSST were analysed using an adjusted Cox proportional hazards model (Supplementary Methods). Restricted means OS analyses were carried out using the pseudovalues method previously described.^22^

## Results

### Study population

**Fig. 1:**
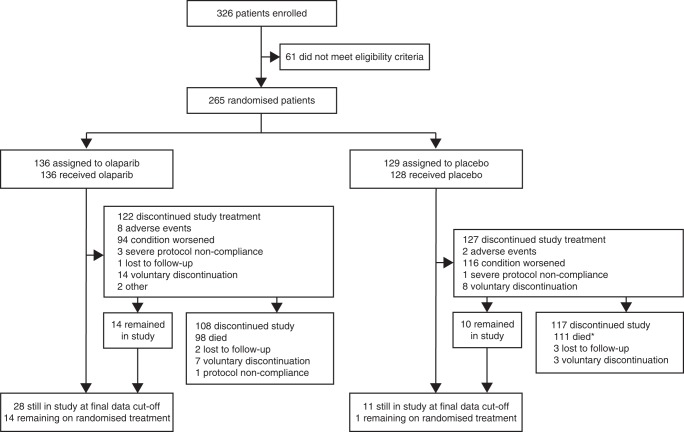
Patient disposition. *One patient was randomly assigned to the placebo group but withdrew consent and withdrew from the study without receiving treatment. This patient subsequently died but is not included in the number of deaths for patients who discontinued the study after being treated with placebo

Of 265 patients randomised in Study 19, 136 were to olaparib and 129 to placebo (Fig. 1). *BRCA*m status was established for 254 of 265 patients (96%; 131 olaparib vs 123 placebo), including 97 for whom *BRCA*m status was known at study entry; 136 patients were classified as *BRCA*m (74 olaparib vs 62 placebo). At the final DCO (9 May 2016), 14 olaparib-arm patients (10%) and 1 placebo-arm patient (1%) were continuing treatment.

Patient demographics and baseline characteristics were generally well balanced for the overall study population and the *BRCA*m and *BRCA*wt subgroups.^5,13^ Patients treated with olaparib for over 2 years had similar characteristics to the overall study population (Table 1, Supplementary Table 1).

**Table 1:** Patient demographics and baseline characteristics

	All patients (*n* = 265)		Patients on treatment ≥ 2 years (*n* = 37)	
	Olaparib (*n* *=* 136)	Placebo (*n* *=* 129)	Olaparib (*n* *=* 32)	Placebo (*n* *=* 5)
Age (years)	58.0 (21‒89)	59.0 (33‒84)	60.0 (43‒80)	59.0 (48‒71)
Ancestry^a^				
Non-Jewish	115 (85)	112 (87)	26 (81)	5 (100)
Jewish	21 (15)	17 (13)	6 (19)	0
Number of previous lines of chemotherapy				
2	59 (43)	63 (49)	14 (44)	3 (60)
3	43 (32)	34 (26)	11 (34)	2 (40)
4	18 (13)	19 (15)	4 (13)	0
≥5	16 (12)	13 (10)	3 (9)	0
Primary tumour location				
Ovary	119 (88)	109 (84)	28 (88)	5 (100)
Fallopian tube or primary peritoneal	17 (13)	20 (16)	4 (13)	0
Time to progression after completion of penultimate platinum-based regimen				
>6 to ≤ 12 months	53 (39)	54 (42)	11 (34)	1 (20)
>12 months	83 (61)	75 (58)	21 (66)	4 (80)
Objective response to most recent platinum-based regimen				
Complete response	57 (42)	63 (49)	18 (56)	4 (80)
Partial response	79 (58)	66 (51)	14 (44)	1 (20)
Secondary debulking ≤ 1 month prior to randomisation	22 (16)	13 (10)	8 (25)	0
Metastatic disease at baseline				
Any site	55 (40)	49 (38)	10 (32)	2 (40)
Lymph nodes	26 (19)	10 (8)	4 (13)	1 (20)
Peritoneum	20 (15)	11 (9)	2 (6)	1 (20)
Hepatic^b^	19 (14)	12 (9)	5 (16)	0

Data are median (range) or *n* (%). Some of these baseline data have been previously reported^5,14^

^a^Ancestry was self-reported

^b^Including gall bladder

### OS and subsequent anticancer therapy

**Fig. 2:**
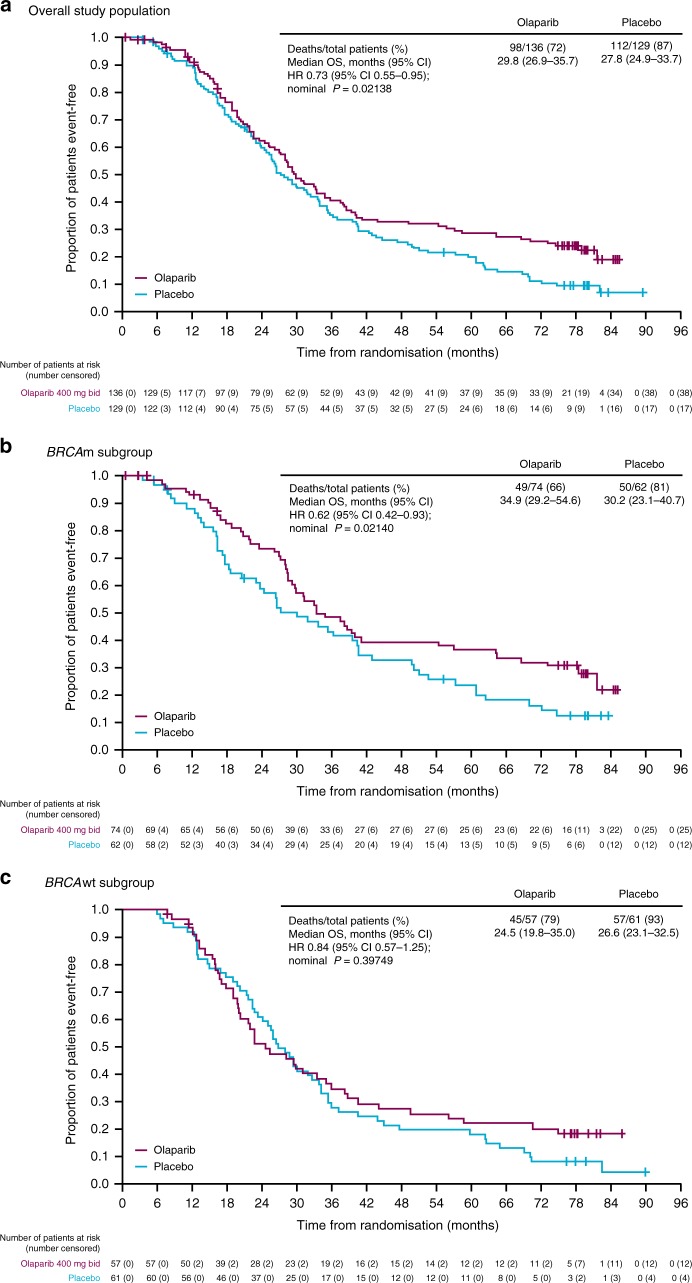
Overall survival in all patients and according to *BRCA* mutation status: **a** overall study population; **b** *BRCA*m subgroup; **c** *BRCA*wt subgroup

The final DCO corresponded to 79% OS data maturity (210 deaths from 265 patients), with a median follow-up of 78.0 months. An apparent advantage in OS for patients randomised to olaparib maintenance monotherapy vs placebo in the total study population (HR 0.73, 95% CI 0.55‒0.95; nominal *P* *=* 0.02138; Fig. 2a) did not meet the threshold defined for statistical significance (*P* *<* 0.0095). A separation in favour of olaparib treatment was seen in the Kaplan–Meier (KM) curves for the overall study population, *BRCA*m and *BRCA*wt subgroups as duration of follow-up increased (Fig. 2). The separation of the KM curves became more apparent after 36 months of follow-up, reflecting the long-term benefit derived for olaparib-treated patients. These data are similar to those reported from the previous DCO (30 September 2015).^14^ Although there was little difference in point estimate medians (29.8 months olaparib vs 27.8 placebo), an exploratory restricted means OS analysis indicated a difference in survival of 6.1 months between treatment arms (95% CI −0.3‒12.6; 41.6 months olaparib vs 35.5 placebo).

Although crossover between treatment arms was not permitted in Study 19, 17 of 129 patients (13.2%) in the placebo group subsequently received PARP inhibitors via other studies. We therefore conducted an exploratory post-hoc OS analysis of 103 olaparib-arm and 92 placebo-arm patients by excluding patients from sites where at least one patient had received subsequent PARP inhibitor treatment. At the final DCO, this resulted in an adjusted OS HR of 0.68 (95% CI 0.49‒0.95; *BRCA*m see Supplementary Results). With the exception of PARP inhibitor treatment, types of subsequent therapy received were similar across both treatment arms (Supplementary Table 2).

### Times to first and second subsequent therapy or death

Exploratory analyses of TFST (87% data maturity, median follow-up 77.4 months) and TSST (85% data maturity, median follow-up 77.1 months) were performed at the final DCO (Supplementary Figure 1). Median TFST and TSST were significantly longer in the olaparib arm in the overall study population, *BRCA*m and *BRCA*wt subgroups.

### Long-term treatment exposure

**Fig. 3:**
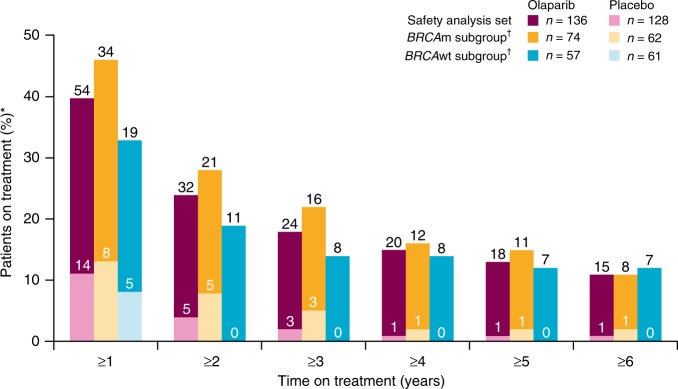
Duration of exposure to treatment. *Patient numbers are shown on the chart. ^†^Ten patients had an unknown *BRCA*m status

At the final DCO (9 May 2016), 15 of 136 patients (11%) had received maintenance olaparib monotherapy for 6 years or more; eight of these had a *BRCA*m and seven were classified as *BRCA*wt (Fig. 3), although one *BRCA*wt patient was subsequently found to carry a somatic *BRCA*m following exploratory biomarker testing.^23^

Twenty-two of 32 olaparib-arm patients (69%) and all five placebo-arm patients who were on treatment for at least 2 years were receiving the full treatment dose (400 mg bid) immediately prior to discontinuation or the end of follow-up. Eight olaparib-arm patients (25%) were at a reduced dose of 200 mg bid, six of whom had their dose reduced prior to 2 years, with the remaining two (6%) receiving 100 mg bid, one of whom had their dose reduced prior to 2 years.

The main reason for discontinuation of study treatment was disease progression (210 of 249 patients; Fig. 1 and Supplementary Figure 2). After 2 years on treatment, the rate of discontinuation of olaparib decreased. Eighteen patients discontinued after this time; nine due to disease progression, four voluntarily, three due to AEs and two due to protocol non-compliance.

At the final DCO, in the full analysis set, mean (standard deviation) compliance with study treatment (assessed using capsule counts) was 96.9% (8.9) in the olaparib arm and 99.0% (3.4) in the placebo arm.

### AE profile

During Study 19, the most common AEs in the olaparib group were early onset of nausea (71%), fatigue/asthenia (63%), vomiting (35%) and diarrhoea (27%), consistent with those previously reported. No new safety findings were observed in the overall study population and safety findings for *BRCA*m patients were similar to those in the overall population (Supplementary Table 3).^5,13,14^ Exposure-adjusted AE rates are presented in the Supplementary Results. Since the study began, serious AEs (SAEs) reported in more than one patient in either treatment group were: anaemia (3 olaparib vs 0 placebo), pancytopenia (2 olaparib [patients subsequently developed myelodysplastic syndrome (MDS) and acute myeloid leukaemia (AML), respectively] vs 0 placebo), constipation (2 olaparib vs 0 placebo), gastritis (0 olaparib vs 2 placebo), small intestinal obstruction (2 olaparib vs 3 placebo), fractured femur (2 olaparib vs 0 placebo) and dyspnoea (2 olaparib vs 0 placebo).

Since the last DCO (30 September 2015), three new SAEs were reported in two patients in the olaparib group (non-cardiac chest pain and aphasia in one patient, who had previously developed brain lesions, and an incarcerated abdominal hernia in the other patient).

A smaller proportion of olaparib-arm patients reported AEs late in treatment (75%) compared with the full study duration (97%), although the prevalence of pruritus, urinary tract infection, dyspnoea and pain in extremity was increased (Table 2a). There were no AEs for which >20% of patients experienced a new episode after 2 years of treatment.

**Table 2:** AEs in Study 19: (**a**) episodes of any grade occurring after ≥ 2 years on treatment in > 7% of olaparib-arm patients in the overall population or *BRCA*m subgroup; (**b**) severity and impact of common AEs on treatment since the start of Study 19

(a)			Overall population			*BRCA*m patients			
Patients on treatment ≥2 years, *n* (%), preferred term			Olaparib, *n* = 32	Placebo, *n* = 5		Olaparib, *n* = 21		Placebo, *n* = 5	
Patients with any AE occurring after 2 years on treatment			24 (75)	4 (80)		16 (76)		4 (80)	
Fatigue/asthenia			6 (19)	0		4 (19)		0	
Constipation			5 (16)	0		4 (19)		0	
Pruritus			5 (16)	0		4 (19)		0	
Urinary tract infection			5 (16)	0		4 (19)		0	
Dizziness			5 (16)	0		3 (14)		0	
Dyspnoea			5 (16)	0		3 (14)		0	
Nausea			4 (13)	0		3 (14)		0	
Abdominal distension			4 (13)	0		3 (14)		0	
Back pain			4 (13)	0		3 (14)		0	
Upper respiratory tract infection			4 (13)	0		3 (14)		0	
Cough			4 (13)	0		2 (10)		0	
Pain in extremity			4 (13)	0		2 (10)		0	
Anaemia^a^			3 (9)	0		3 (14)		0	
Bone pain			3 (9)	0		3 (14)		0	
Headache			3 (9)	1 (20)		2 (10)		1 (20)	
Peripheral swelling			3 (9)	0		2 (10)		0	
Blood creatinine increased			3 (9)	0		1 (5)		0	
Diarrhoea			3 (9)	0		1 (5)		0	
Sinusitis			3 (9)	0		1 (5)		0	
Vomiting			3 (9)	0		1 (5)		0	
Abdominal pain			2 (6)	1 (20)		2 (10)		1 (20)	
Alopecia			2 (6)	0		2 (10)		0	
Cystitis			2 (6)	0		2 (10)		0	
Ecchymosis			2 (6)	0		2 (10)		0	
Osteoarthritis			2 (6)	0		2 (10)		0	
Pancytopenia			2 (6)	0		2 (10)		0	
Pyrexia			2 (6)	0		2 (10)		0	
Sensory disturbance			2 (6)	0		2 (10)		0	
Sleep disorder			2 (6)	0		2 (10)		0	
(b)	Nausea		Vomiting			Fatigue/asthenia		Anaemia^a^	
	Olaparib	Placebo	Olaparib		Placebo	Olaparib	Placebo	Olaparib	Placebo
*n*	136	128	136		128	136	128	136	128
Patients with AEs	96 (71)	46 (36)	48 (35)		18 (14)	86 (63)	59 (46)	31 (23)	9 (7)
Patients whose first incidence occurred after >6 months on treatment	12 (9)	4 (3)	11 (8)		4 (3)	18 (13)	4 (3)	10 (7)	0
Total episodes^b^	129	58	92		20	118	72	40	10
Grade 1	102 (79)	46 (79)	66 (72)		12 (60)	69 (58)	58 (81)	5 (13)	6 (60)
Grade 2	24 (19)	12 (21)	23 (25)		7 (35)	37 (31)	10 (14)	24 (60)	3 (30)
Grade 3 or 4	3 (2)	0	3 (3)		1 (5)	12 (10)	4 (6)	11 (28)	1 (10)
Treatment interrupted	9 (7)	1 (2)	18 (20)		1 (5)	8 (7)	2 (3)	4 (10)	0
Treatment dose reduced	5 (4)	0	4 (4)		1 (5)	9 (8)	1 (1)	8 (20)	1 (10)
Treatment discontinued	1 (1)	1 (2)	0		0	0	0	0	0
AE resolved	105 (81)	46 (79)	90 (98)		17 (85)	73 (62)	35 (49)	28 (70)	7 (70)
Treatment required	56 (43)	10 (17)	21 (23)		3 (15)	5 (4)	3 (4)	30 (75)	2 (20)
Median time to onset of first event, days	4.0	13.0	52.0		64.5	28.0	29.0	29.0	92.0
Median duration of first event, months	2.7	0.8	0.1		0.1	3.0	3.3	2.8	0.5

Arbitrary cut-off corresponding to three or more patients in the overall population, or two or more patients in the *BRCA*m subgroup

All AEs reported after 2 years of treatment are included irrespective of whether this was the first incidence of a specific AE; incidences that began before 2 years, but that continued past 2 years on treatment are not included; no AEs were reported after 2 years by more than one patient in the placebo arm

*AE* adverse event

^a^Includes patients with anaemia, haemoglobin decreased, red blood cell count decreased and haematocrit decreased

^b^Patients could experience more than one episode of the AE

Of the 264 patients who received study treatment, 209 deaths had occurred at the time of the final DCO (98 olaparib vs 111 placebo). In the investigators’ opinion, the vast majority of patients in both treatment groups died because of progression of their ovarian cancer (188 patients). Since the study began, two patients, both in the olaparib arm and both with a *BRCA*m, experienced AEs that resulted in death. One of these deaths had occurred at the time of the last DCO; this patient died due to AEs of haemorrhagic stroke and thrombocytopenia, deemed to be treatment related.^14^ The other death was due to previously reported AML; this AE occurred 14 days after discontinuation of study treatment and the patient, who had received olaparib for over 4 years, died approximately 1 year and 9 months later. This patient previously was reported to have a SAE of pancytopenia while on study treatment. This was the only case of AML reported in Study 19; two patients were reported with MDS, as described previously^14^ (see Supplementary Results). All three patients had previously received two lines of platinum-based chemotherapy. Four olaparib-treated patients developed new primary malignancies: adenocarcinoma of the colon, ductal carcinoma in situ, papillary thyroid cancer and squamous cell carcinoma of the oral cavity (see Supplementary Results). Two cases of pneumonitis were reported during the study, one in the olaparib arm <3 months into treatment, and one in the placebo arm, both of grade 1 severity.

Of those patients who received olaparib, throughout the whole study 53 (39%) had dose interruptions (47 [35%] for AEs) and 59 (43%) had dose reductions (35 [26%] for AEs). In the placebo arm, 23 patients (18%) had dose interruptions (13 [10%] for AEs) and 29 patients (23%) had dose reductions (5 [4%] for AEs; *BRCA*m see Supplementary Results). Since the last DCO, no new AEs have led to discontinuation of study treatment.^14^ Overall, AEs leading to discontinuation of study treatment were reported for eight (6%) olaparib-arm and two (2%) placebo-arm patients (see Supplementary Results); no single AE was the reason for discontinuation of more than one patient in either treatment arm. All AEs leading to treatment discontinuation were considered related to study treatment in the investigator’s opinion, and have been previously reported.^5,13,14^

### Characterisation of common AEs

We further characterised the common AEs of nausea, vomiting, fatigue/asthenia and anaemia in Study 19 by determining their severity and outcome (Table 2b). These events were generally low grade, did not require treatment modification, and only two patients (one per treatment arm) discontinued treatment due to common AEs. Anaemia was the most common haematological toxicity, occurring in 23 and 7% of olaparib- and placebo-treated patients, respectively. At study entry, 20 patients (15%) in the olaparib arm had anaemia vs 14 (11%) in the placebo arm and 18 olaparib-treated patients (13%) received a blood transfusion during the trial, (1 [1%] placebo). Data for *BRCA*m patients were consistent with those for the overall population (Supplementary Tables 5 and 6).

**Fig. 4:**
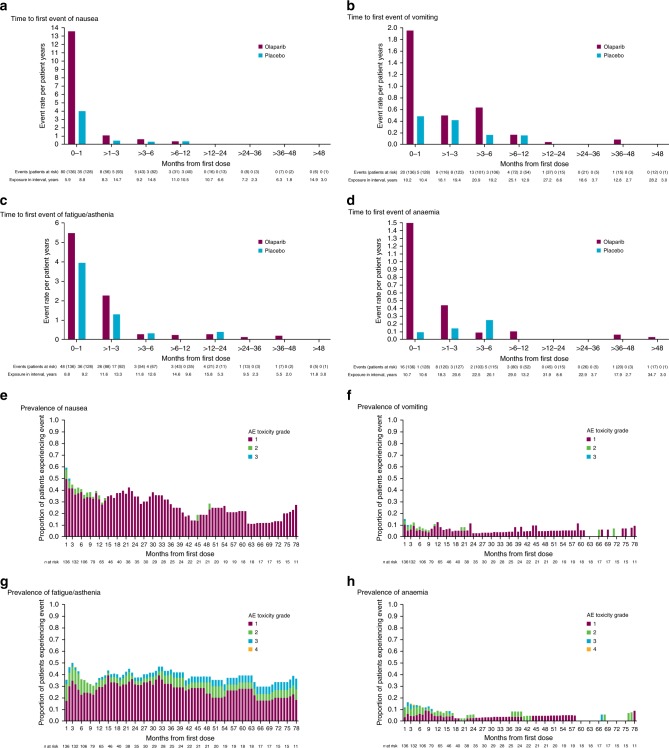
Characterisation of common AEs: time to first event (Event rate = number of first events/exposure during time interval. Note that *y* axes scales are different between parts **a**, **b**, **c** and **d**) of **a** nausea, **b** vomiting, **c** fatigue/asthenia and **d** anaemia^†^, and prevalence by month and grade of **e** nausea, **f** vomiting, **g** fatigue/asthenia and **h** anaemia^†^ in olaparib-treated patients. ^†^Includes patients with anaemia, haemoglobin decreased, red blood cell count decreased and haematocrit decreased

Patients who experienced common AEs typically did so for the first time within a few months of the start of treatment and it was rare for common AEs to initially develop after >6 months on olaparib (event rates < 0.3 per patient year; Fig. 4, Supplementary Figure 3 [*BRCA*m patients]). Prevalence plots for the common AEs show the proportion of patients at risk (receiving treatment or within their 30 day follow-up period) who experienced each AE during a specific month. This is irrespective of AE start date, so AEs with a long duration are represented over multiple months; note that when there are only a few patients on treatment the proportion experiencing an AE can appear to be high (Fig. 4, Supplementary Figure 4 [*BRCA*m patients]).

## Discussion

We report the final analyses of long-term efficacy and tolerability data from Study 19, a Phase II trial assessing olaparib maintenance monotherapy (400 mg bid, capsule formulation) in patients with platinum-sensitive, recurrent high-grade serous ovarian cancer in response to their most recent platinum-based chemotherapy regimen, representing the longest follow-up from any PARP inhibitor trial reported. Our findings suggest an OS advantage for patients receiving maintenance olaparib vs placebo, an effect not yet observed for any chemotherapy regimen or other maintenance therapies in the recurrent ovarian cancer setting.^3,24^ It should be noted that although Study 19 was not designed or powered to show a statistically significant difference in OS, this OS advantage with olaparib is consistent with previously published analyses.^5,13,14^

The apparent OS benefit seen with olaparib appears to be driven by long-term responders, with a separation in KM survival curves between treatment arms seen after approximately 3 years.^5,13^ This is supported by an exploratory restricted means OS analysis, carried out to assess average life-expectancy over the whole survival curve, taking into account differences in hazard ratio over time. Although consistent with previously reported data showing that *BRCA*m patients had the greatest PFS benefit from olaparib,^13^ these long-term OS results indicate that a subset of patients within the *BRCA*wt group also experience durable long-term benefit from maintenance olaparib monotherapy. It is likely that these patients have homologous recombination repair (HRR) deficiencies (germline or somatic) resulting in synthetic lethality with exposure to olaparib. Approximately 50% of patients with high-grade serous ovarian cancer are thought to have a deficiency in HRR, opening up maintenance therapy for a significant number of women with recurrent ovarian cancer.^25^ The challenge is to identify which *BRCA*wt patients are most likely to benefit from a PARP inhibitor. It has been recently reported that a positive homologous recombination deficiency (HRD) score was associated with long-term response ( > 2 years) to olaparib.^12^ Additionally, a biomarker analysis of patients in Study 19 who remained on treatment for ≥ 6 years has shown at least 5 of 15 patients were classified as *BRCA*wt. This *BRCA*wt group included patients with other HRR pathway gene mutations or a positive HRD score, as well as a patient with no positive candidate predictive biomarker test results.^23^ Other baseline characteristics were broadly similar between the full study population and those on long-term treatment, although an increased proportion of patients on olaparib for at least 2 years had undergone secondary debulking surgery shortly prior to randomisation in Study 19. The long-term survivors in this study represent a highly selected subpopulation. Although it is likely that factors such as patient heterogeneity and the number of subsequent lines of treatment following progression may have contributed to the OS of patients, it should be noted that in this randomised trial, 15 (11%) patients were on olaparib for > 6 years, compared with only one (0.8%) placebo recipient. It remains to be established why these patients achieved such a durable response to olaparib.

Following progression, OS may be influenced by subsequent therapy with platinum agents, bevacizumab and/or PARP inhibitors. Crossover was not permitted in our study; however, 13% of placebo-arm patients subsequently received a PARP inhibitor via other clinical trials. This could have caused confounding of the OS data and, consistent with this, we show an improved OS HR for olaparib vs placebo when excluding patients from sites where at least one patient received subsequent treatment with a PARP inhibitor, confirming similar findings from an earlier interim analysis.^26^ This approach, excluding sites, was preferred to a similar method excluding just crossover patients as it was felt to lessen the potential confounding of removing only well-performing placebo patients and better retain the benefits of randomisation. As PARP inhibitors become more commonly utilised and widely available, the feasibility of showing a statistically significant OS benefit is decreasing; no future trials will be able to have as little crossover as Study 19 and, given the results of this and other maintenance studies with PARP inhibitors, placebo-controlled trials would no longer be ethical in this population.

Exploratory data for TFST and TSST reported here show significant benefits with maintenance olaparib monotherapy compared with placebo for the overall population and for the *BRCA*m and *BRCA*wt subgroups, consistent with previous analyses.^13,14^ A clear separation between the olaparib and placebo KM survival curves can be seen extending past 6 years of follow-up. Since no tumour assessments were carried out in Study 19 after the primary DCO (30 June 2010), TFST represents a reasonable long-term surrogate for PFS, which was analysed with a median of 5.6 months’ follow-up. TFST and TSST data are consistent with the observed advantage in PFS and OS for olaparib-arm patients and show a clinically meaningful increase in time between chemotherapy regimens, while also suggesting an extended efficacy benefit with olaparib persisting beyond the next line of therapy.^27^

The long-term exposure seen in Study 19 is unprecedented for a PARP inhibitor; consistently across *BRCA*m and *BRCA*wt subgroups, 10% of patients experienced a durable benefit from olaparib maintenance monotherapy for over 6 years. Almost 25% of patients received olaparib for at least 2 years (compared with 4% of placebo-arm patients), considerably longer than the expected median PFS of 4–6 months in this population.^5–7^ A higher proportion of patients who received olaparib long term had at least three prior lines of chemotherapy compared with those who received placebo. The rate of discontinuation of olaparib for any reason decreased after 2 years, suggesting patients who reach this milestone are likely to receive continued benefit from maintenance olaparib. It should be noted that patients continued treatment long-term despite taking 16 large capsules per day; a more patient-friendly formulation of olaparib, requiring only 4 tablets per day, is now available.^10,11^

No new safety signals were identified since the previous analysis and data reported here indicate that the tolerability profile of olaparib is compatible with long-term treatment. Very few patients discontinued treatment due to AEs in Study 19; 6 and 2% in the olaparib and placebo arms, respectively. The majority of patients receiving olaparib long term were on full dose immediately prior to the end of treatment, which suggests enduring dose modifications are not required to maintain long-term tolerability.

AEs of MDS and AML were rare in Study 19 and were reported in both treatment arms. The incidence in Study 19 was broadly consistent with that seen in SOLO2.^6^ The MDS, AML and new primary malignancy AEs that occurred in Study 19 were unrelated to duration of exposure to olaparib.

In line with previously published olaparib data,^16–18^ the most common AEs in Study 19 were low-grade nausea, fatigue/asthenia and vomiting, with anaemia the most common haematological AE. These AEs usually occurred early and rarely first developed after >6 months on olaparib or placebo. Only one patient discontinued olaparib due to one of these common AEs (nausea). Prevalence data show that the proportion of patients who experienced vomiting or anaemia during any specific month was low. More patients at a given time experienced nausea and fatigue/asthenia, however, there was no increase in prevalence of these AEs with long-term treatment. Due to the increased length of time that patients spent on olaparib compared with placebo, it is expected that more AEs will be observed in the olaparib arm. Exposure-adjusted AE data (Supplementary Table 4), which show the number of AEs per year on treatment, demonstrate more limited differences between the two treatment groups for the common AEs and show a higher rate of fatigue/asthenia in the placebo arm; this suggests that the prevalence of this AE is similar irrespective of treatment arm, and that fatigue/asthenia may not be caused by olaparib.

These safety data should be considered in the context of toxicity profiles associated with palliative chemotherapy, which is commonly prescribed to patients at disease progression. There are many potential adverse effects (AEs) associated with chemotherapy, which can be cumulative, limiting the length of time patients can remain on treatment.^8^ Currently, the reporting of AEs with PARP inhibitors is based on an approach developed for AEs associated with chemotherapy and arguably, new methods of AE reporting, including patient reported AEs, may be more appropriate for characterising the intermittent AEs typically observed with PARP inhibitors. In this study, intermittent AEs could have been reported differently by individual investigators, either as a single event of long duration or multiple events of short duration. This inconsistency may have resulted in higher prevalence being observed for events such as nausea, and fatigue/asthenia that had a long median duration (and may have been intermittent during this time). The generally low-grade, non-cumulative nature of the common AEs observed with olaparib in Study 19 supports the approach that they can be routinely managed by physicians through dose modifications when required and symptomatic treatment with standard procedures, such as antiemetics or occasional blood transfusions, although regular haematological monitoring is recommended for patients receiving olaparib.^10,11,28^

Various factors should be considered when weighing the value of long-term maintenance therapy with a PARP inhibitor against platinum-based chemotherapy alone followed by further lines of treatment for recurrent disease at symptomatic progression. Maintenance olaparib may not be effective in all patients, and is often associated with low-grade intermittent toxicity that can usually be controlled with simple supportive measures. However, its efficacy and generally favourable long-term tolerability profile, coupled with previously reported data showing no detrimental impact on patients’ HRQoL during treatment,^19^ makes olaparib a viable maintenance treatment option following response to platinum-based chemotherapy, which should be discussed with patients. Maintenance therapy with olaparib capsules prolonged PFS in Study 19,^5^ and results from the ongoing Phase III SOLO2 trial of maintenance therapy with the tablet formulation of olaparib also showed a significant increase in median PFS for *BRCA*m patients, compared with placebo.^6^ Long-term follow-up from SOLO2 and other ongoing trials will help further elucidate the efficacy and tolerability profiles of olaparib maintenance monotherapy and ultimately identify those patients with ovarian cancer most likely to receive long-term benefit from maintenance therapy with olaparib.

In addition, patient preference data showing the trade-off that women with recurrent ovarian cancer who are receiving maintenance PARP inhibitor therapy are willing to make in terms of gains in PFS and OS vs avoiding toxicity are also needed. Survey results indicate that most women with recurrent ovarian cancer required an increase in PFS of at least 5 months to make treatment worthwhile,^29^ and patient preference data suggest that women with recurrent ovarian cancer would accept a shorter PFS in order to avoid severe adverse effects associated with chemotherapy (e.g., nausea, vomiting).^30^ A delay in the time to subsequent chemotherapy is also likely to be of importance to patients, with TFST and TSST significantly prolonged with olaparib vs placebo in both Study 19 and SOLO2.^6^

## Conclusions

The final analysis from Study 19 is the largest long-term survival follow-up data for a PARP inhibitor and suggests an OS advantage with olaparib maintenance monotherapy for patients with platinum-sensitive, recurrent high-grade serous ovarian cancer. Although the threshold for statistical significance was not met, a numerical OS advantage was seen, irrespective of *BRCA*m status, despite considerable crossover. This finding is supported by significant improvements in PFS, TFST and TSST with maintenance olaparib compared with placebo in *BRCA*m or *BRCA*wt women. Almost 25% of patients in Study 19 received olaparib for at least 2 years and over 10% continued on treatment for 6 years or more, demonstrating a prolonged, clinically meaningful benefit derived from olaparib maintenance therapy, which is unprecedented in patients with recurrent ovarian cancer. The capsule formulation of olaparib was well tolerated in Study 19; the majority of AEs in both the overall population and *BRCA*m subgroup were low grade and manageable with dose modifications or simple supportive treatments and no new tolerability signals were identified with long-term treatment. Taken together, these data support the use of olaparib maintenance monotherapy as long-term treatment for patients with platinum-sensitive, recurrent high-grade serous ovarian cancer with or without a *BRCA*m.

## Supplementary information

Supplemental_revised clean
