# Supplementary material for: Long-term efficacy, tolerability and overall survival in patients with platinum-sensitive, recurrent high-grade serous ovarian cancer treated with maintenance olaparib capsules following response to chemotherapy
Source: Br J Cancer. 2018 Oct 24;119(9):1075–85. doi: 10.1038/s41416-018-0271-y (PMC6219499; doi:10.1038/s41416-018-0271-y)
Supplement: Supplementary file 1 — Supplemental_revised clean [file 41416_2018_271_MOESM1_ESM.docx]

**SUPPLEMENTARY MATERIAL**

**Long-term efficacy, tolerability and overall survival in patients with platinum-sensitive, recurrent high-grade serous ovarian cancer treated with maintenance olaparib capsules following response to chemotherapy**

Michael Friedlander^1^, Ursula Matulonis^2^, Charlie Gourley^3^, Andreas du Bois^4^, Ignace Vergote^5^, Gordon Rustin^6^, Clare Scott^7^, Werner Meier^8^, Ronnie Shapira-Frommer^9^, Tamar Safra^10^, Daniela Matei^11^, Vadim Shirinkin^12^, Frédéric Selle^13^, Anitra Fielding^14^, Elizabeth S. Lowe^15^, Emma L. McMurtry^14^, Stuart Spencer^14^, Philip Rowe^14^, Helen Mann^14^, David Parry^14^, Jonathan Ledermann^16^

*^1^University of New South Wales Clinical School, Prince of Wales Hospital, Randwick, Australia;
^2^Dana-Farber Cancer Institute, Boston, MA, USA;
^3^Cancer Research UK Edinburgh Centre, Western General Hospital, Edinburgh, UK; ^4^Kliniken Essen Mitte, Essen, Germany;
^5^University of Leuven, Leuven Cancer Institute, Leuven, European Union;
^6^Mount Vernon Hospital, Northwood, UK;
^7^Royal Melbourne Hospital, Parkville, Australia;*  *^8^University of Düsseldorf, Düsseldorf, Germany;
^9^Chaim Sheba Medical Center, Tel Hashomer, Israel;
^10^Tel Aviv Sourasky Medical Center, Tel Aviv, Sackler School of Medicine, Tel Aviv University, Tel Aviv, Israel;
^11^Northwestern University Feinberg School of Medicine, Chicago, IL, USA;
^12^Orenburg Regional Clinical Oncological Dispensary, Orenburg, Russia;
^13^Groupe Hospitalier Diaconesses Croix Saint-Simon, Paris, and GINECO group, France;
^14^AstraZeneca, Combridge, UK;
^15^AstraZeneca, Gaithersburg, MD, USA;
^16^University College London Cancer Institute, London, UK*

**Supplementary Methods**

*Study design and population*

Platinum-sensitivity was defined as no disease progression within 6 months of the last dose of the penultimate line of platinum-based chemotherapy. Patients were in complete or partial response to their most recent platinum-based chemotherapy regimen according to Response Evaluation Criteria in Solid Tumours (RECIST) version 1.0 or Gynecological Cancer InterGroup criteria.

*BRCA mutation testing*

Germline *BRCA*m status was established using the Integrated BRACAnalysis^®^ assay (Myriad Genetics Laboratories, Salt Lake City, UT, USA),^1^ with DNA extracted from blood samples obtained prior to randomisation, or was reported from previous local germline *BRCA* testing. Tumour *BRCA*m status was assessed using prospectively collected tumour samples and the Foundation Medicine T5 panel (Foundation Medicine, Cambridge, MA, USA), a previously validated next-generation sequencing protocol.^2^ Patients with no known *BRCA*m, or a variant of unknown significance, were classified as *BRCA*wt.^3,4^

*Treatments*

Treatment was interrupted for any National Cancer Institute’s Common Terminology Criteria for Adverse Events (CTCAE) grade 3 or 4 event considered related to treatment and was restarted at a reduced dose (200 or 100 mg bid) if the event resolved, entirely or to grade 1, within 4 weeks. If the event did not resolve within a 4-week period, or the patient had already had two treatment interruptions, they were withdrawn from the trial. Patients could continue receiving study treatment after progression if the investigator deemed the patient was benefiting, but could not crossover between treatment arms.

*Statistical analyses*

Previous analyses of OS have been conducted at 38% (data cut-off [DCO]: 31 October 2011; two-sided α=0.1%), 58% (DCO: 26 November 2012; two-sided α=3%), and 77% data maturity (DCO: 30 September 2015; two-sided α=0.95%).^3-5^ Previous analyses of TFST (80% and 86% data maturity) and TSST (74% and 84% data maturity) have been conducted.^3,4^ A multiple testing strategy was only pre-specified for OS in the whole study population and not in the *BRCA*m or *BRCA*wt subgroups. A Cox proportional hazards model was used to analyse OS, TFST and TSST and was adjusted by ancestry (Jewish vs non-Jewish), time to progression from completion of penultimate platinum-based regimen (6–12 vs >12 months) and response to most recent platinum-based regimen (complete vs partial). Control of type I error was not defined for the exploratory endpoints TFST and TSST. The analysis set for TFST, TSST and safety included all patients who received at least one dose of study medication.

**Supplementary Results**

*Adjusted overall survival and subsequent cancer therapy for patients with a BRCA mutation*

In the *BRCA*m subgroup 14 placebo-arm patients (22.6%) received subsequent PARP inhibitor treatment. This left 96 patients (57 olaparib; 39 placebo) for inclusion in an exploratory post-hoc OS analysis, excluding patients from sites where at least one patient had received subsequent PARP inhibitor treatment, that resulted in an adjusted HR of 0.49 (95% CI 0.28‒0.85). Patients with a *BRCA*m received similar subsequent therapies to those in the overall population (Supplementary Table 2).

*Dose modifications for patients with a BRCA mutation*

For the *BRCA*m subgroup, 31 patients (42%) in the olaparib arm had dose interruptions (26 [35%] due to AEs) and 33 (45%) had dose reductions (18 [24%] due to AEs). In the placebo arm, 13 patients (21%) had dose interruptions (6 [10%] due to AEs) and 14 patients (23%) had dose reductions (2 [3%] due to AEs). Six of the eight olaparib arm patients who discontinued treatment due to an AE had a *BRCA*m, two of whom had received two prior lines of chemotherapy, with the remaining four patients having received three prior lines of chemotherapy. Two of the *BRCA*m patients discontinued after more than 2 years on treatment. Neither of the two placebo arm patients who discontinued treatment due to an AE had a *BRCA*m.

*Exposure adjusted adverse event rates*

Exposure adjusted AE rates, which show the number of AEs per year on treatment, are presented in Supplementary Table 4. A bigger difference between treatment arms was seen for the *BRCA*m subgroup, and while there were only small differences between treatment arms for fatigue/asthenia, rates of nausea were higher for olaparib-treated patients.

*Reports of myelodysplastic syndrome and new primary malignancies*

The first case of myelodysplastic syndrome (MDS) was diagnosed in a *BRCA*wt patient over 30 days after discontinuation of olaparib, and was considered possibly related to study treatment; it was recorded as a secondary cause of death, with the primary reason related to ovarian cancer. The patient received olaparib for less than 1 year, and had also previously been reported with an SAE of pancytopenia while on study treatment. The second case of MDS was in a placebo-arm patient with a *BRCA*m who received study treatment for over 3 years.

Four olaparib-treated patients developed new primary malignancies: adenocarcinoma of the colon and ductal carcinoma in situ each occurred in one patient with a *BRCA*m and developed after less than 2 years on treatment; one *BRCA*wt patient developed papillary thyroid cancer, and one patient with unknown *BRCA*m status developed squamous cell carcinoma of the oral cavity, both after more than 5 years on treatment.

*Discontinuations because of adverse events*

AEs leading to discontinuation occurred in eight (6%) olaparib-arm patients and two (2%) placebo-arm patients. Four of the olaparib-arm patients had received two prior lines of chemotherapy, and four had received three prior lines; both placebo-arm patients had received two prior lines of chemotherapy. Three of the patients who discontinued olaparib due to an AE did so after 2 years of treatment; one due to grade 4 pancytopenia and grade 1 pharyngitis, one due to grade 2 bronchiectasis, and one with a new primary squamous cell carcinoma of the oral cavity.

Supplementary Table 1. Patient demographics and baseline characteristics

|  | **All patients** | | | | | | **Patients on treatment ≥2 years** | | | | | | |
| --- | --- | --- | --- | --- | --- | --- | --- | --- | --- | --- | --- | --- | --- |
|  | **Patients with *BRCA*m (n=136)^†^** | | | | **Patients with *BRCA*wt* (n=118)^†^** | | **Patients with *BRCA*m (n=26)^†^** | | | **Patients with *BRCA*wt* (n=11)^†^** | | | |
|  | Olaparib (*n* = 74) | | | Placebo (*n* = 62) | Olaparib (*n* = 57) | Placebo (*n* = 61) | Olaparib (*n* = 21) | Placebo (*n* = 5) | Olaparib (*n* = 11) | | | Placebo (*n* = 0) |  |
| Age (years) | 57.5 (38‒89) | | | 55.0 (33‒84) | 62.0 (21‒80) | 63.0 (49‒79) | 60.0  (43‒80) | 59.0  (48‒71) | 63.0  (44‒79) | | | – |  |
| Ancestry^‡^  Non-Jewish  Jewish | 60 (81)  14 (19) | | | 48 (77)  14 (23) | 51 (89)  6 (11) | 58 (95)  3 (5) | 16 (76)  5 (24) | 5 (100)  0 | 10 (91)  1 (9) | | | –  – |  |
| Number of previous lines of chemotherapy | | | | | | | | | | |  |  |  |
| 2  3  4  ≥5 | 26 (35)  28 (38)  9 (12)  11 (15) | | | 28 (45)  18 (29)  10 (16)  6 (10) | 32 (56)  14 (25)  6 (11)  5 (9) | 35 (57)  14 (23)  9 (15)  3 (5) | 9 (43)  7 (33)  4 (19)  1 (5) | 3 (60)  2 (40)  0  0 | 5 (45)  4 (36)  1 (9)  1 (9) | | | –  –  –  – |  |
| Primary tumour location | | | | | | | | | | |  |  |  |
| Ovary | 65 (88) | | | 54 (87) | 50 (88) | 49 (80) | 18 (86) | 5 (100) | 10 (91) | | | – |  |
| Fallopian tube or primary peritoneal | 9 (12) | | | 8 (13) | 7 (12) | 12 (20) | 3 (14) | 0 | 1 (9) | | | – |  |
| Time to progression after completion of penultimate platinum-based regimen | | | | | | | | | | |  |  |  |
| >6 to ≤12 months | 28 (38) | | | 26 (42) | 23 (40) | 24 (39) | 7 (33) | 1 (20) | 4 (36) | | | – |  |
| >12 months | 46 (62) | | | 36 (58) | 34 (60) | 37 (61) | 14 (67) | 4 (80) | 7 (64) | | | – |  |
| Objective response to most recent platinum-based regimen | | | | | | | | | | |  |  |  |
| Complete response | | 36 (49) | | 34 (55) | 20 (35) | 25 (41) | 12 (57) | 4 (80) | 6 (55) | | | – |  |
| Partial response | | 38 (51) | | 28 (45) | 37 (65) | 36 (59) | 9 (43) | 1 (20) | 5 (45) | | | – |  |
| Secondary debulking ≤1 month prior to randomisation | | 12 (16) | | 7 (11) | 10 (18) | 6 (10) | 6 (29) | 0 | 2 (18) | | | – |  |
| Metastatic disease at baseline | | | | | | | | | | |  |  |  |
| Any site | | | 27 (36) | 16 (26) | 24 (42) | 30 (49) | 5 (24) | 2 (40) | 5 (45) | | | – |  |
| Lymph nodes | | | 14 (19) | 5 (8) | 11 (19) | 4 (7) | 2 (10) | 1 (20) | 2 (18) | | | – |  |
| Peritoneum | | | 11 (15) | 6 (10) | 8 (14) | 5 (8) | 0 | 1 (20) | 2 (18) | | | – |  |
| Hepatic^§^ | | | 11 (15) | 5 (8) | 7 (12) | 7 (11) | 2 (10) | 0 | 3 (27) | | | – |  |

Data are median (range) or n (%). Some of these baseline data have been previously reported.^3^ **BRCA*wt subgroup included patients with no detected *BRCA*m and patients with *BRCA*m of unknown significance. ^†^Data were not available for all randomised patients. ^‡^Ancestry was self-reported. ^§^Including gall bladder.

Supplementary Table 2. Subsequent anticancer therapy received by patients in the full analysis set and *BRCA*m subgroup

|  | **Overall population** | | ***BRCA*m patients** | | ***BRCA*wt patients** | |
| --- | --- | --- | --- | --- | --- | --- |
| **Patients, n (%)** | **Olaparib *n* = 136** | **Placebo *n* = 129** | **Olaparib *n* = 74** | **Placebo *n* = 62** | **Olaparib *n* = 57** | **Placebo *n* = 61** |
| Line of subsequent therapy |  |  |  |  |  |  |
| 1st | 91 (67) | 111 (86) | 47 (64) | 55 (89) | 41 (72) | 52 (85) |
| 2nd | 61 (45) | 84 (65) | 33 (45) | 39 (63) | 26 (46) | 42 (69) |
| 3rd | 48 (35) | 63 (49) | 26 (35) | 29 (47) | 21 (37) | 31 (51) |
| 4th | 27 (20) | 36 (28) | 15 (20) | 17 (27) | 11 (19) | 17 (28) |
| ≥5th | 17 (13) | 18 (14) | 10 (14) | 9 (15) | 6 (11) | 8 (13) |
| Platinum-containing regimen | 69 (51) | 71 (55) | 35 (47) | 35 (56) | 32 (56) | 34 (56) |
| Bevacizumab containing regimen | 14 (10) | 15 (12) | 8 (11) | 5 (8) | 5 (9) | 8 (13) |
| Other anticancer regimen (non-platinum or bevacizumab) | 64 (47) | 86 (67) | 38 (51) | 41 (66) | 24 (42) | 42 (69) |
| PARP inhibitor | 0 | 17 (13) | 0 | 14 (23) | 0 | 3 (5) |

Supplementary Figure 1. TFST and TSST in all patients and according to *BRCA* mutation status

**A** TFST in overall study population


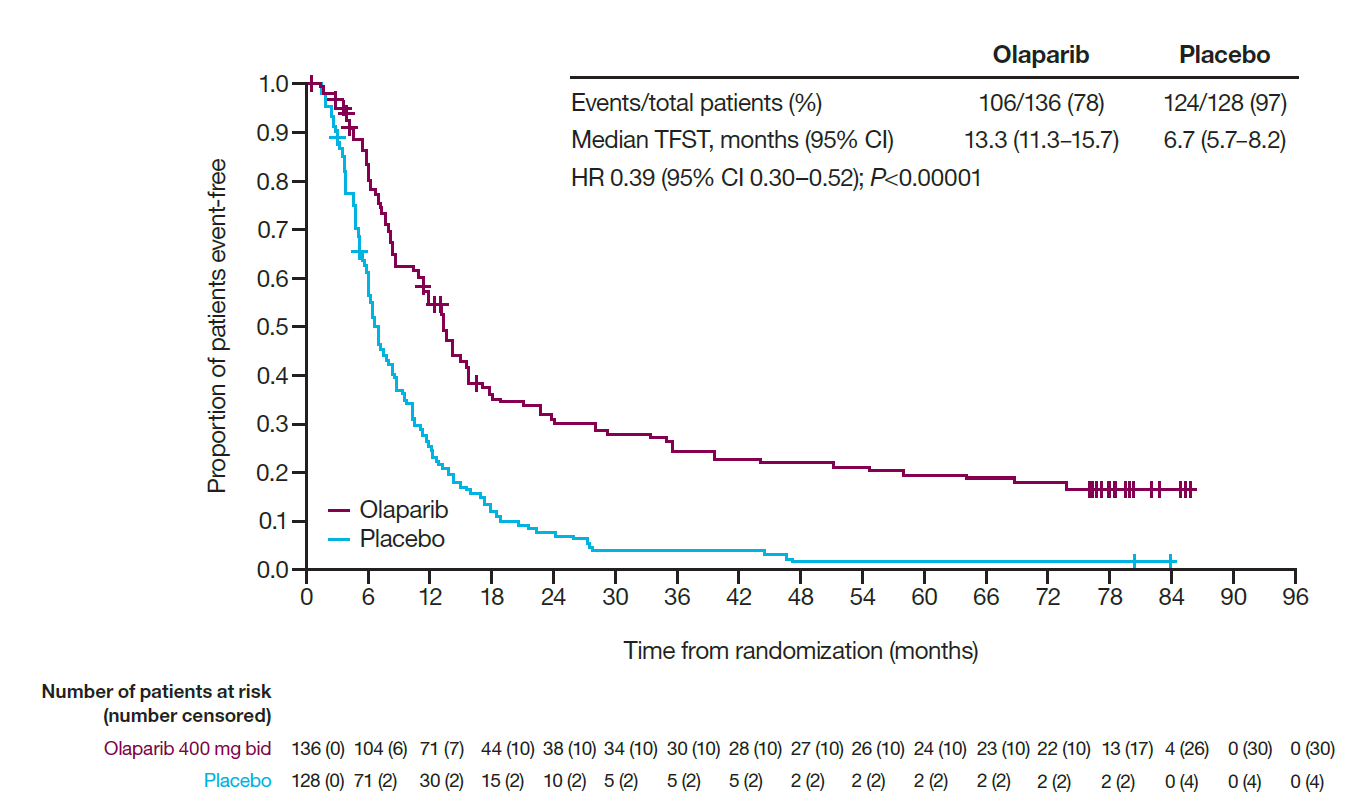


**B** TFST in *BRCA*m subgroup


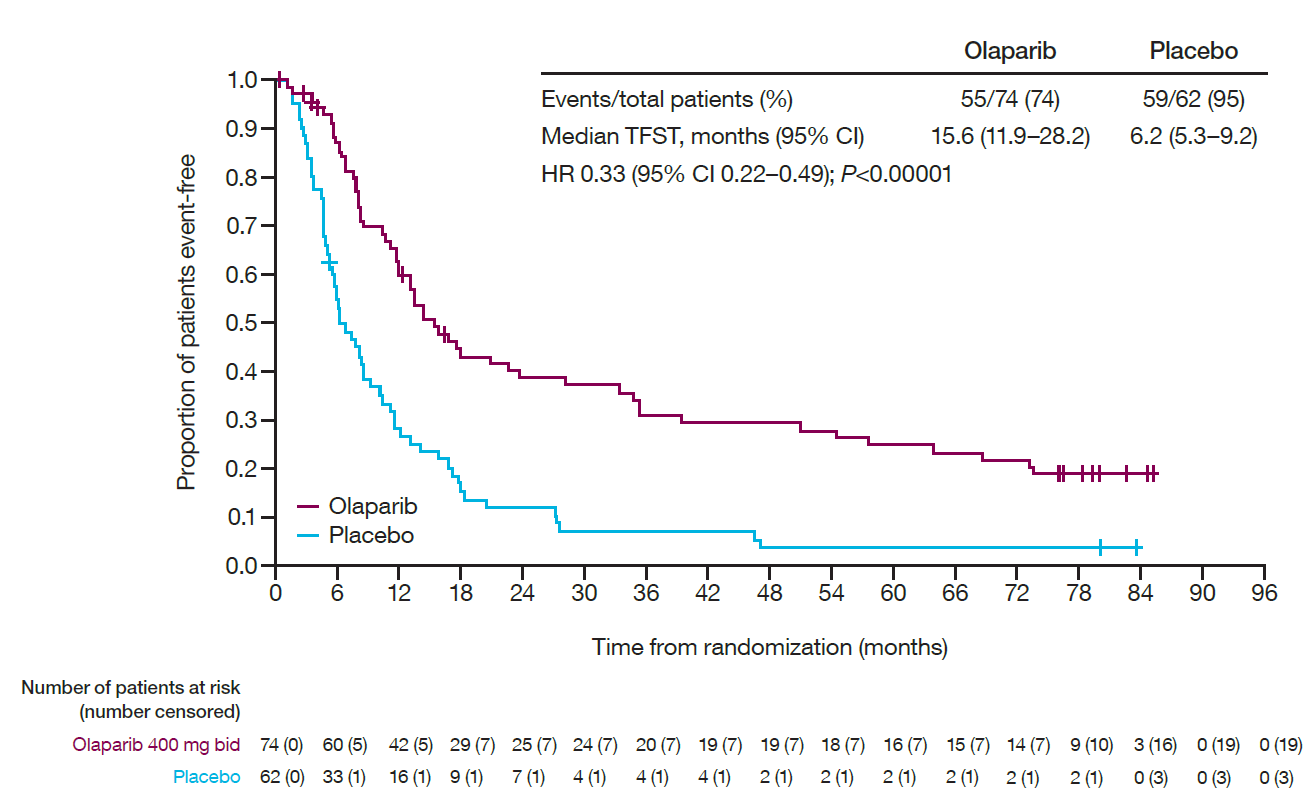


**C** TFST in *BRCA*wt subgroup


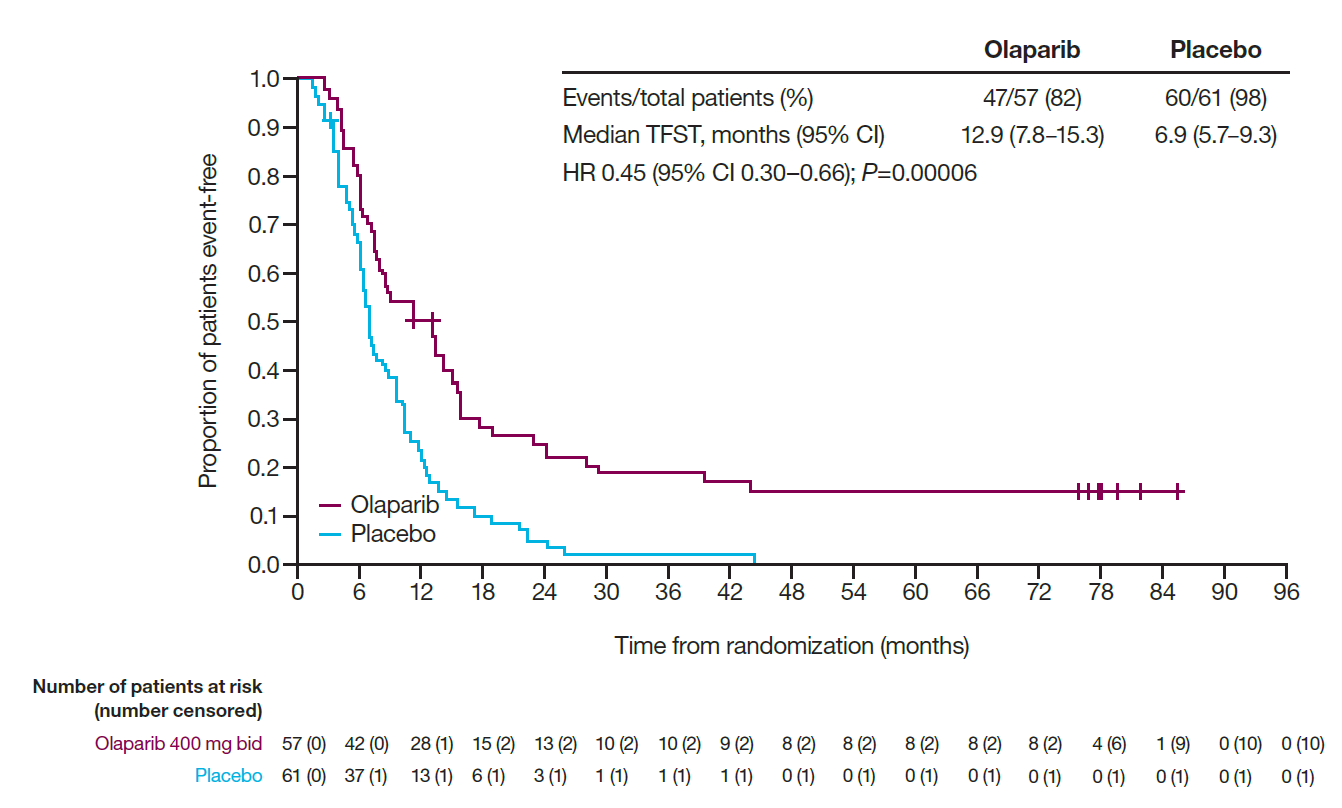


**D** TSST in overall study population


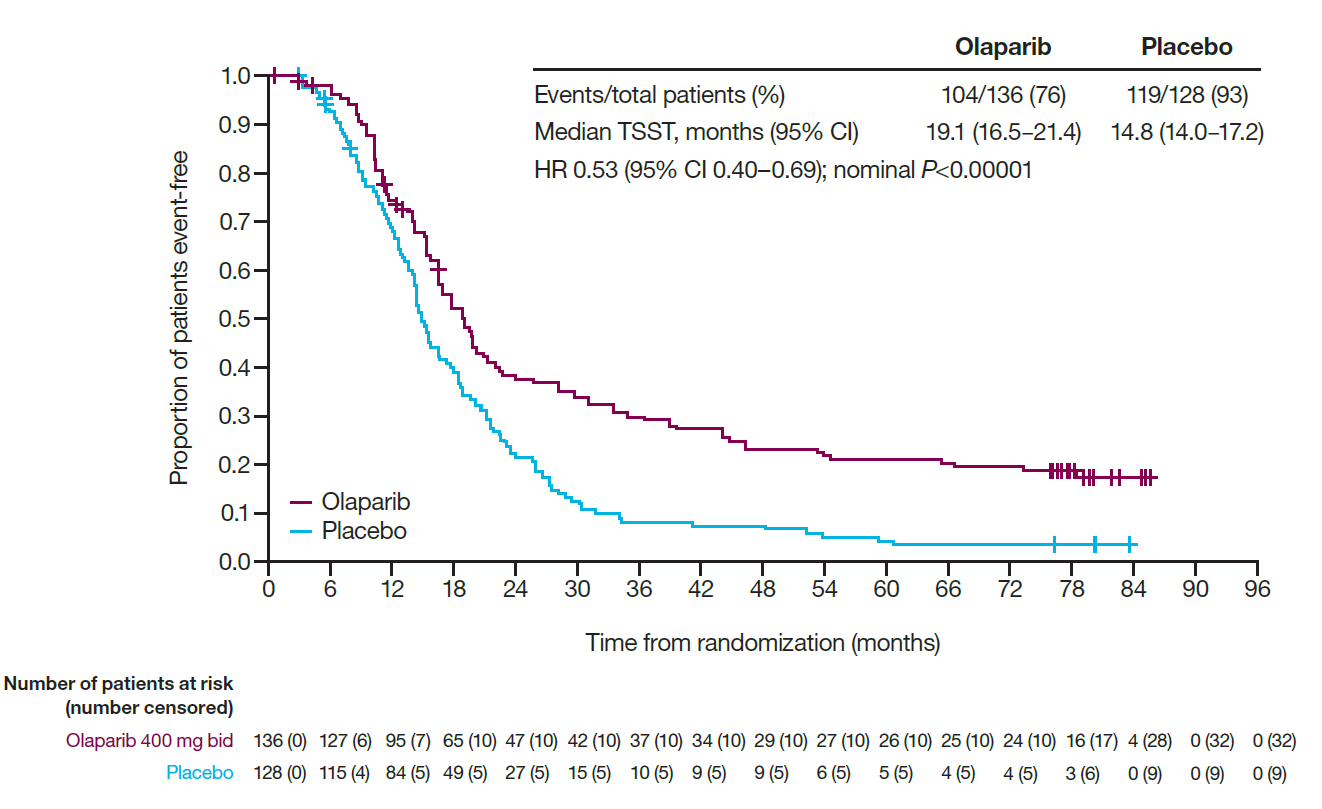


**E** TSST in *BRCA*m subgroup


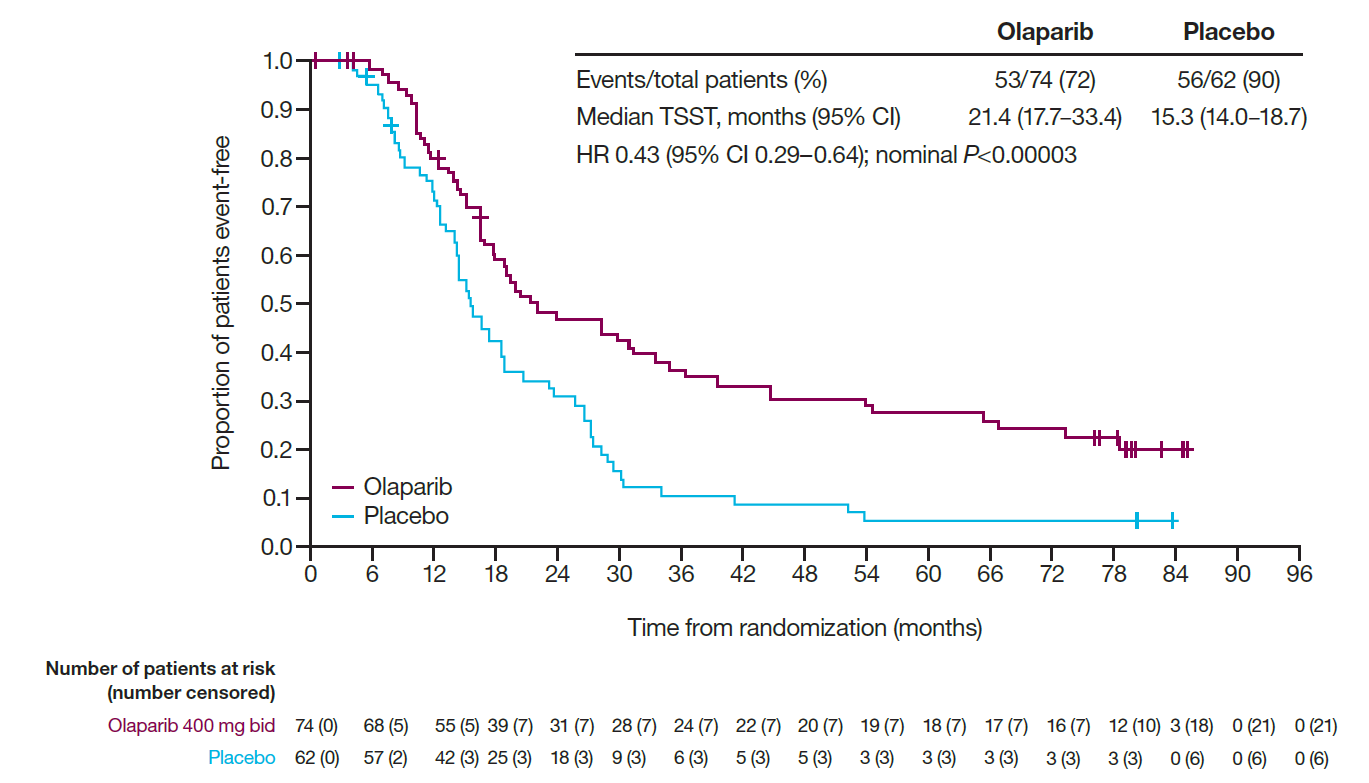


**F** TSST in *BRCA*wt subgroup


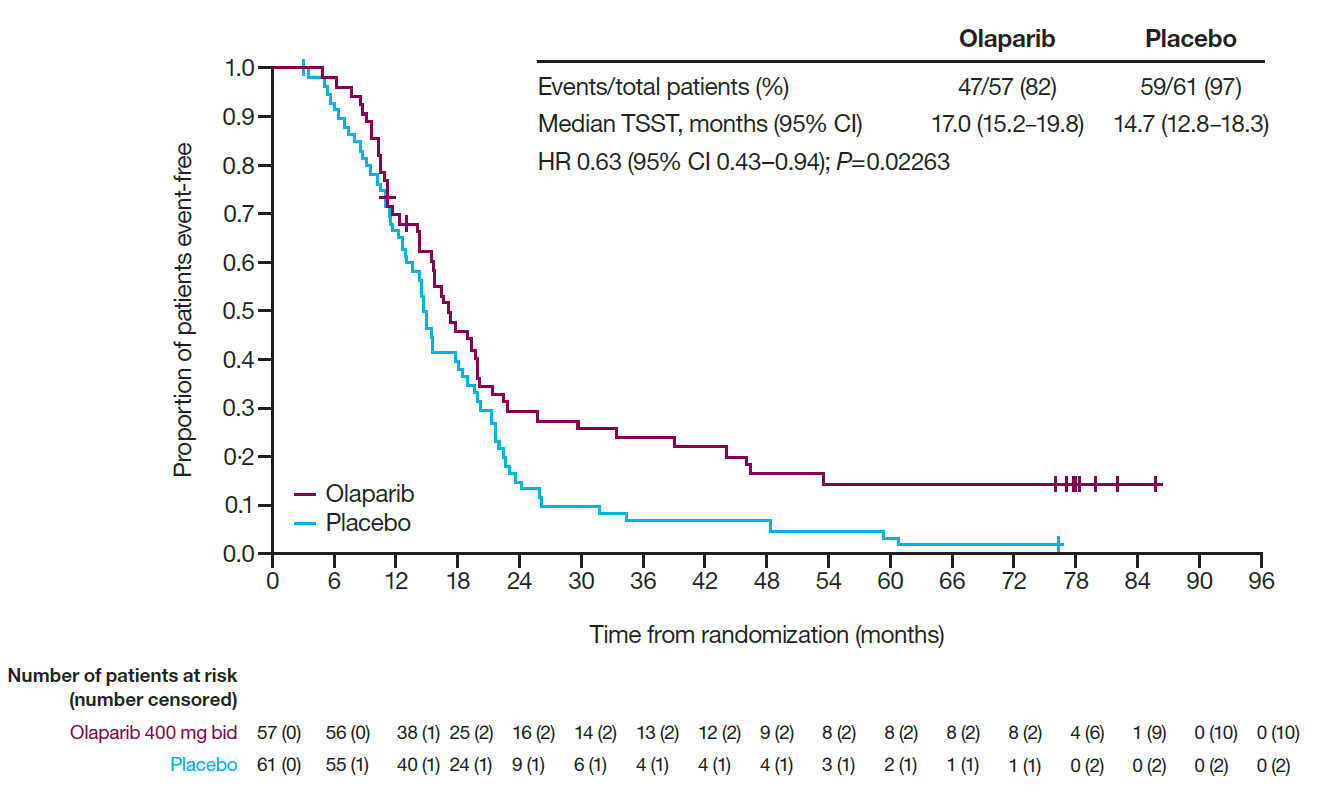


Supplementary Figure 2. Time to discontinuation of study treatment


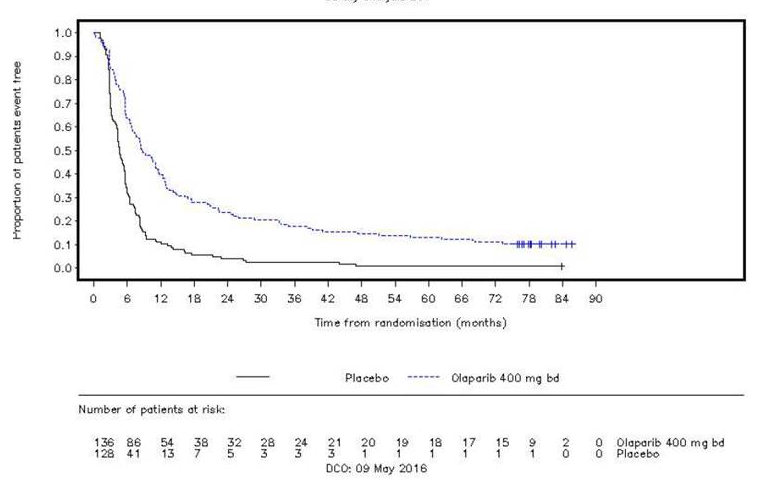


Supplementary Table 3. AEs of all grades (frequency >10%), of grade ≥3 (frequency ≥5%), and of haematological interest for the overall population and *BRCA*m subgroup

|  | **Overall population** | | | | ***BRCA*m patients** | | | | |  |
| --- | --- | --- | --- | --- | --- | --- | --- | --- | --- | --- |
| ***n* (%)** | **Olaparib**  ***n* = 136** | **Placebo**  ***n* = 128** | **Olaparib**  ***n* = 136** | **Placebo**  ***n* = 128** | | **Olaparib**  ***n* = 74** | **Placebo**  ***n* = 62** | **Olaparib**  ***n* = 74** | **Placebo**  ***n* = 62** | |
| **Preferred term** | **All grades** | | **Grade ≥3** | | | **All grades** | | **Grade ≥3** | | |
| **Total episodes** | **1796** | **1002** | **102** | **45** | | **1062** | **523** | **64** | **20** | |
| **Patients with any AE** | **132 (97)** | **119 (93)** | **59 (43)** | **28 (22)** | | **72 (97)** | **58 (94)** | **31 (42)** | **11 (18)** | |
| Nausea | 96 (71) | 46 (36) | 3 (2) | 0 | | 54 (73) | 20 (32) | 1 (1) | 0 | |
| Fatigue/asthenia | 86 (63) | 59 (46) | 12 (9) | 4 (3) | | 49 (66) | 28 (45) | 7 (9) | 1 (2) | |
| Vomiting | 48 (35) | 18 (14) | 3 (2) | 1 (1) | | 28 (38) | 5 (8) | 2 (3) | 0 | |
| Diarrhoea | 37 (27) | 31 (24) | 3 (2) | 3 (2) | | 22 (30) | 12 (19) | 2 (3) | 1 (2) | |
| Abdominal pain | 35 (26) | 34 (27) | 3 (2) | 4 (3) | | 18 (24) | 18 (29) | 0 | 2 (3) | |
| Constipation | 30 (22) | 14 (11) | 1 (1) | 0 | | 16 (22) | 7 (11) | 0 | 0 | |
| Anaemia* | 31 (23) | 9 (7) | 10 (7) | 1 (1) | | 20 (27) | 5 (8) | 6 (8) | 1 (2) | |
| Decreased appetite | 29 (21) | 17 (13) | 0 | 0 | | 15 (20) | 6 (10) | 0 | 0 | |
| Headache | 29 (21) | 17 (13) | 0 | 1 (1) | | 14 (19) | 11 (18) | 0 | 1 (2) | |
| Dyspepsia | 27 (20) | 11 (9) | 0 | 0 | | 14 (19) | 4 (6) | 0 | 0 | |
| Back pain | 25 (18) | 14 (11) | 4 (3) | 0 | | 16 (22) | 9 (15) | 3 (4) | 0 | |
| Upper abdominal pain | 25 (18) | 11 (9) | 0 | 1 (1) | | 14 (19) | 5 (8) | 0 | 0 | |
| Arthralgia | 24 (18) | 18 (14) | 1 (1) | 0 | | 12 (16) | 10 (16) | 1 (1) | 0 | |
| Cough | 24 (18) | 13 (10) | 0 | 0 | | 11 (15) | 7 (11) | 0 | 0 | |
| Dysgeusia | 22 (16) | 8 (6) | 0 | 0 | | 14 (19) | 4 (6) | 0 | 0 | |
| Dizziness | 21 (15) | 9 (7) | 0 | 0 | | 13 (18) | 3 (5) | 0 | 0 | |
| Nasopharyngitis | 21 (15) | 14 (11) | 0 | 0 | | 11 (15) | 4 (6) | 0 | 0 | |
| Abdominal distension | 21 (15) | 11 (9) | 0 | 0 | | 12 (16) | 6 (10) | 0 | 0 | |
| Upper respiratory tract infection | 19 (14) | 8 (6) | 0 | 0 | | 13 (18) | 6 (10) | 0 | 0 | |
| Dyspnoea | 18 (13) | 8 (6) | 2 (2) | 0 | | 5 (7) | 3 (5) | 1 (1) | 0 | |
| Urinary tract infection | 16 (12) | 7 (6) | 0 | 1 (1) | | 10 (14) | 4 (6) | 0 | 1 (2) | |
| Pyrexia | 14 (10) | 4 (3) | 1 (1) | 0 | | 9 (12) | 1 (2) | 1 (1) | 0 | |
| Neuropathy peripheral | 12 (9) | 3 (2) | 0 | 0 | | 9 (12) | 1 (2) | 0 | 0 | |
| Depression | 11 (8) | 9 (7) | 0 | 0 | | 8 (11) | 6 (10) | 0 | 0 | |
| Neutropenia | 7 (5) | 5 (4) | 5 (4) | 1 (1) | | 5 (7) | 3 (5) | 3 (4) | 1 (2) | |
| Abdominal pain, lower | 7 (5) | 10 (8) | 0 | 0 | | 3 (4) | 7 (11) | 0 | 0 | |
| Hot flush | 5 (4) | 16 (13) | 0 | 0 | | 5 (7) | 12 (19) | 0 | 0 | |
| Thrombocyto-penia | 5 (4) | 3 (2) | 1 (1) | 0 | | 3 (4) | 2 (3) | 1 (1) | 0 | |

*Includes patients with anaemia, haemoglobin decreased, red blood cell count decreased and haematocrit decreased

Supplementary Table 4. Frequency of any and common AEs when adjusted for exposure to study treatment in Study 19

|  | **Overall population** | | | | ***BRCA*m patients** | | | |
| --- | --- | --- | --- | --- | --- | --- | --- | --- |
|  | **Olaparib**  ***n* = 136** | **Placebo**  ***n* = 128** | **Olaparib**  ***n* = 136** | **Placebo**  ***n* = 128** | **Olaparib**  ***n* = 74** | **Placebo**  ***n* = 62** | **Olaparib**  ***n* = 74** | **Placebo**  ***n* = 62** |
| **Event rate per 1000 years on study treatment** | **All grades** | | **Grade ≥3** | | **All grades** | | **Grade ≥3** | |
| **Patients with any AE** | **9612** | **8300** | **400** | **382** | **12643** | **7601** | **334** | **263** |
| Nausea | 1308 | 740 | 13 | 0 | 1999 | 563 | 7 | 0 |
| Vomiting | 298 | 226 | 13 | 12 | 330 | 106 | 14 | 0 |
| Fatigue/asthenia | 965 | 1037 | 57 | 47 | 903 | 835 | 55 | 21 |
| Anaemia* | 165 | 110 | 44 | 12 | 195 | 107 | 44 | 21 |

*Includes patients with anaemia, haemoglobin decreased, red blood cell count decreased and haematocrit decreased

Supplementary Table 5. Severity and impact on treatment of common AEs in *BRCA*m patients in Study 19

|  | **Nausea** | | **Vomiting** | | **Fatigue/asthenia** | | **Anaemia*** | |
| --- | --- | --- | --- | --- | --- | --- | --- | --- |
| ***n* (%)** | **Olaparib** | **Placebo** | **Olaparib** | **Placebo** | **Olaparib** | **Placebo** | **Olaparib** | **Placebo** |
| *n* | 74 | 62 | 74 | 62 | 74 | 62 | 74 | 62 |
| Patients with AEs | 54 (73) | 20 (32) | 28 (38) | 5 (8) | 49 (66) | 28 (45) | 20 (27) | 5 (8) |
| Patients whose first incidence occurred after >6 months on treatment | 9 (12) | 1 (2) | 6 (8) | 1 (2) | 13 (18) | 3 (5) | 7 (9) | 0 |
| Total episodes^†^ | 72 | 27 | 60 | 5 | 69 | 36 | 27 | 6 |
| Grade 1  Grade 2  Grade 3 or 4 | 62 (86)  9 (13)  1 (1) | 23 (85)  4 (15)  0 | 44 (73)  14 (23)  2 (3) | 4 (80)  1 (20)  0 | 38 (55)  24 (35)  7 (10) | 31 (86)  4 (11)  1 (3) | 2 (7)  18 (67)  7 (26) | 4 (67)  1 (17)  1 (17) |
| Treatment interrupted | 5 (7) | 1 (4) | 14 (23) | 0 | 5 (7) | 1 (3) | 3 (11) | 0 |
| Treatment dose reduced | 3 (4) | 0 | 2 (3) | 0 | 5 (7) | 0 | 5 (19) | 1 (17) |
| Treatment discontinued | 0 | 0 | 0 | 0 | 0 | 0 | 0 | 0 |
| AE resolved | 60 (83) | 20 (74) | 59 (98) | 4 (80) | 44 (64) | 17 (47) | 19 (70) | 4 (67) |
| Treatment required | 28 (39) | 6 (22) | 14 (23) | 1 (20) | 4 (6) | 0 | 20 (74) | 0 |
| Median time to onset of first event, days | 4 | 8 | 48 | 32 | 27 | 29 | 36 | 84 |
| Median duration of first event, months | 3.5 | 0.8 | 0.1 | 0.1 | 3.4 | 3.4 | 2.4 | 0.5 |

*Includes patients with anaemia, haemoglobin decreased, red blood cell count decreased and haematocrit decreased. ^†^Patients could experience more than one episode of the AE.

Supplementary Table 6. *BRCA*m patients with anaemia at study entry and requiring a blood transfusion during Study 19

| **n (%)** | **Olaparib**  ***n* = 74** | **Placebo**  ***n* = 62** |
| --- | --- | --- |
| Anaemia at study entry | 12 (16) | 7 (11) |
| Transfusion during study | 9 (12) | 0 |

Supplementary Figure 3. Time to first event* of A) nausea, B) vomiting, C) fatigue/asthenia, and D) anaemia^†^ in patients with a *BRCA*m

**A** Time to first event of nausea


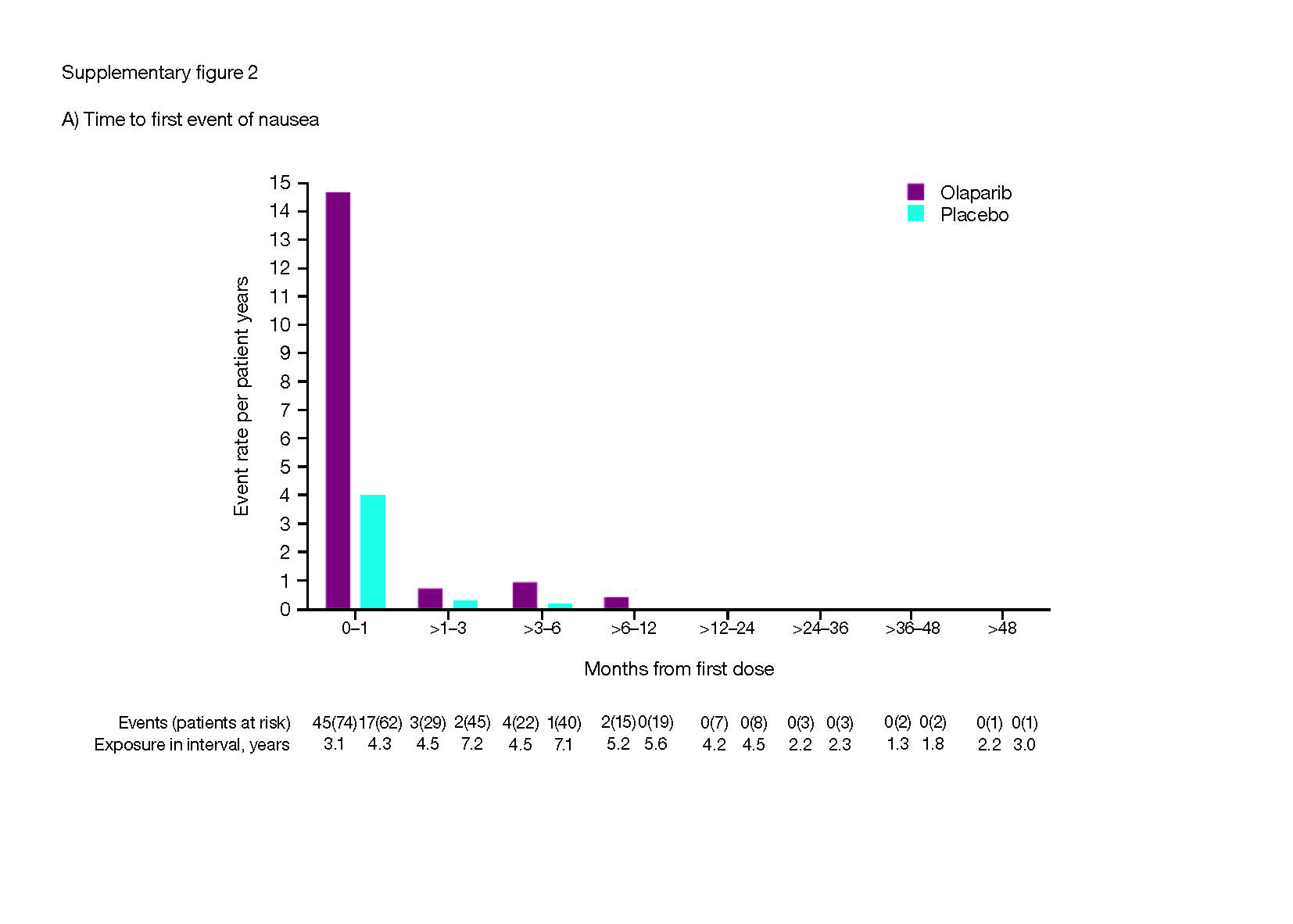


**B** Time to first event of vomiting


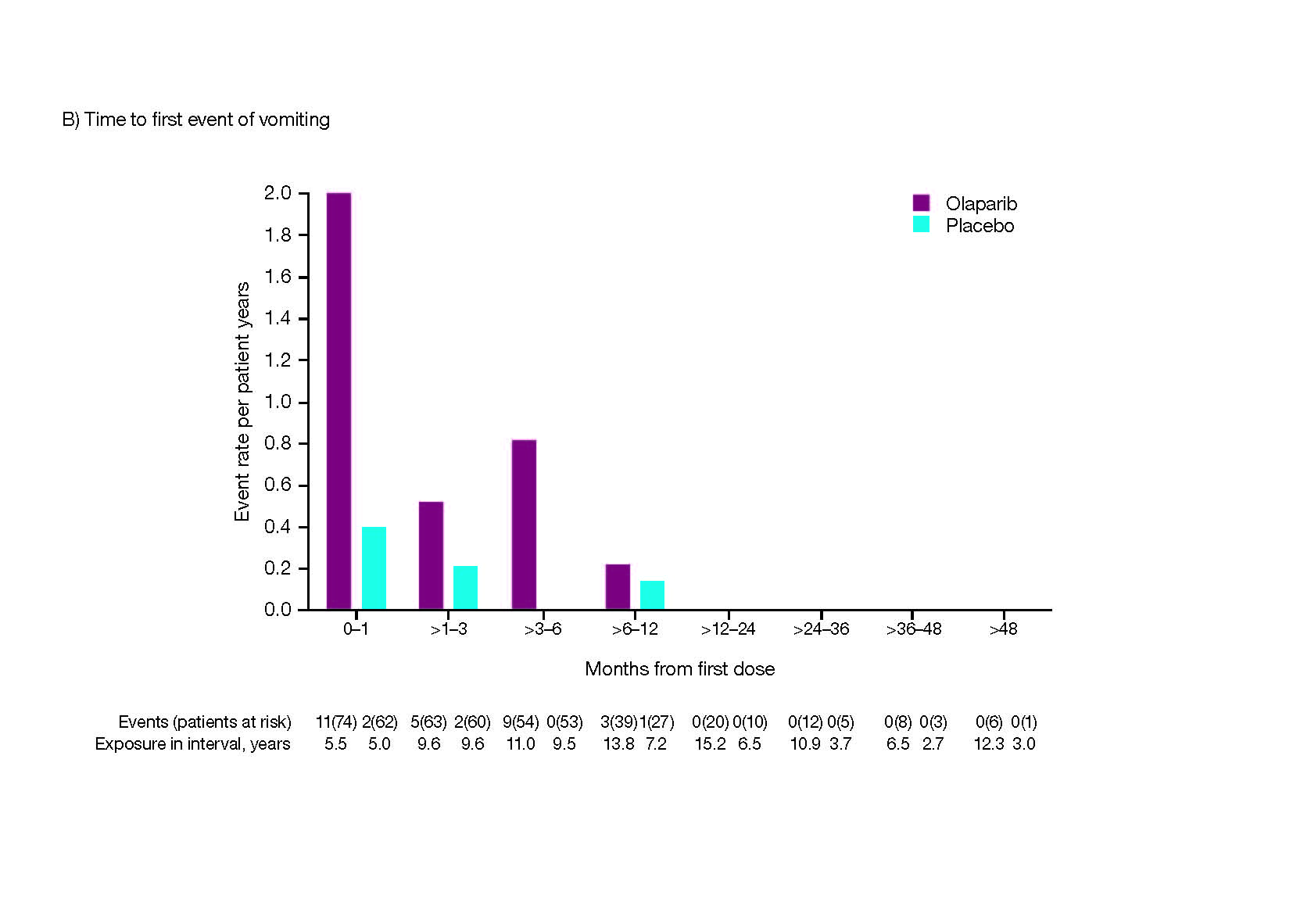


**C** Time to first event of fatigue/asthenia


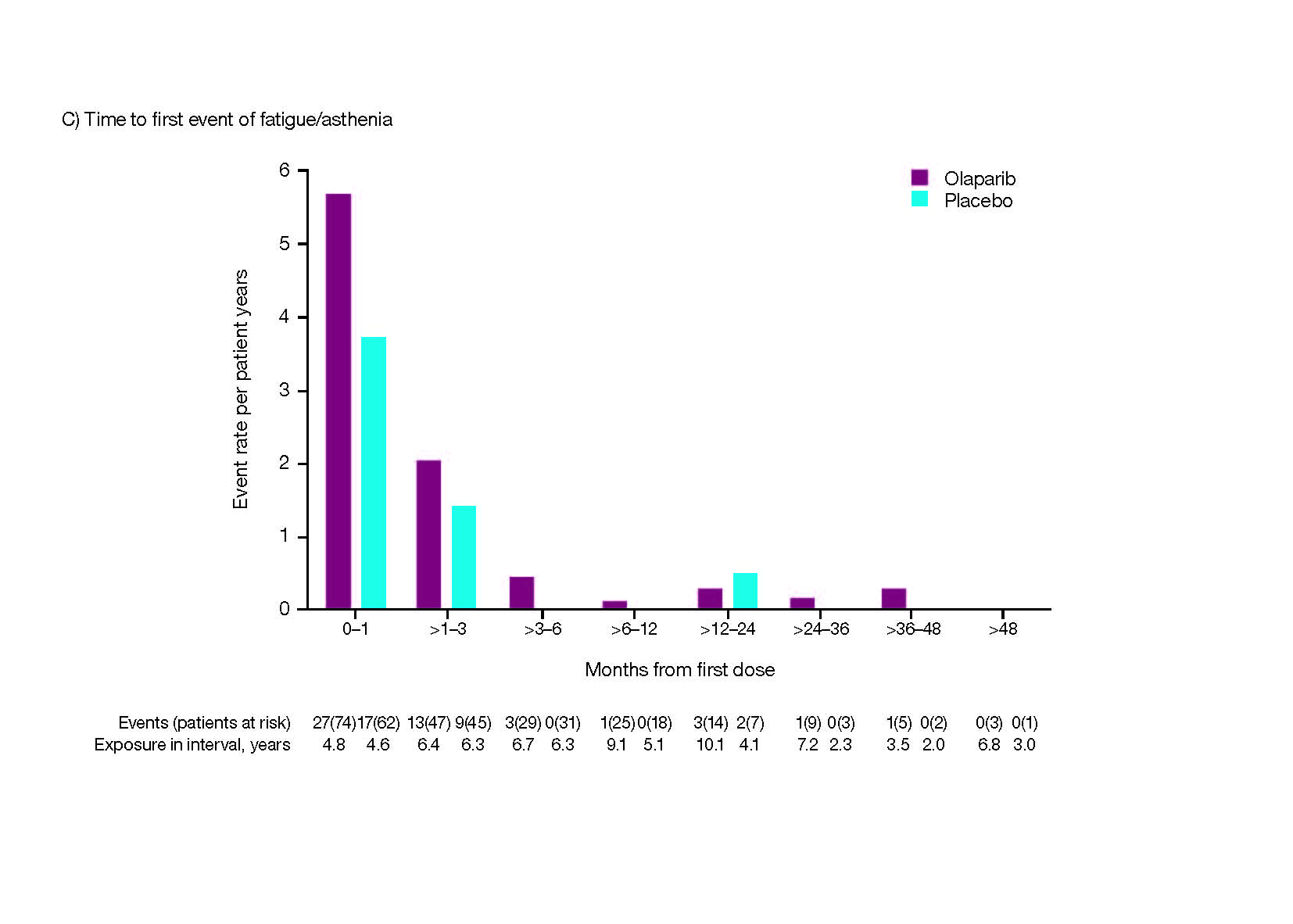


**D** Time to first event of anaemia^†^


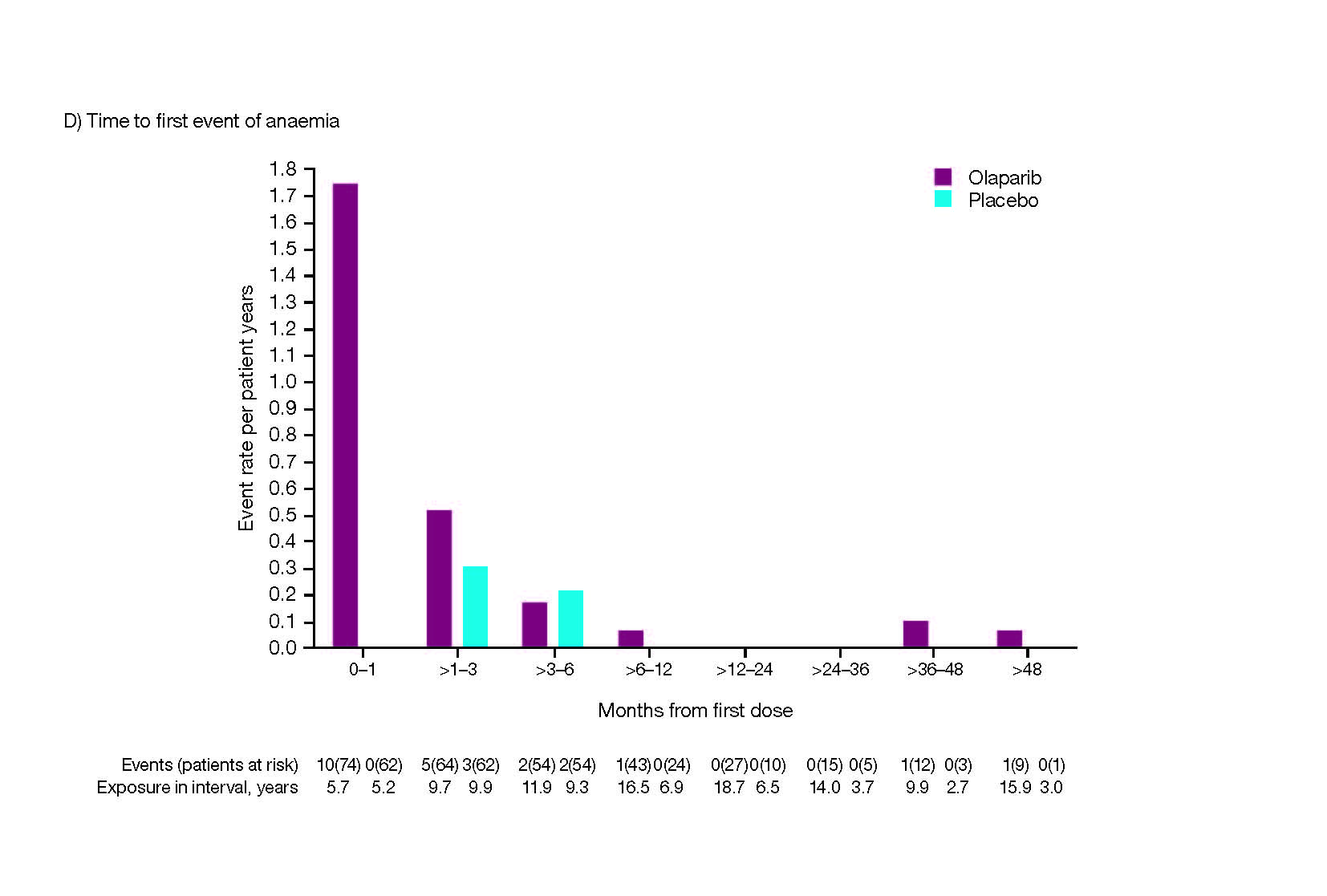


*Event rate = number of first events / exposure during time interval. Note that y-axes scales are different between parts A, B, C and D. ^†^Includes patients with anaemia, haemoglobin decreased, red blood cell count decreased and haematocrit decreased

Supplementary Figure 4. Prevalence by month and grade of common AEs in olaparib-treated patients with a *BRCA*m: A) nausea, B) vomiting, C) fatigue/asthenia, and D) anaemia*

**A** Prevalence of nausea


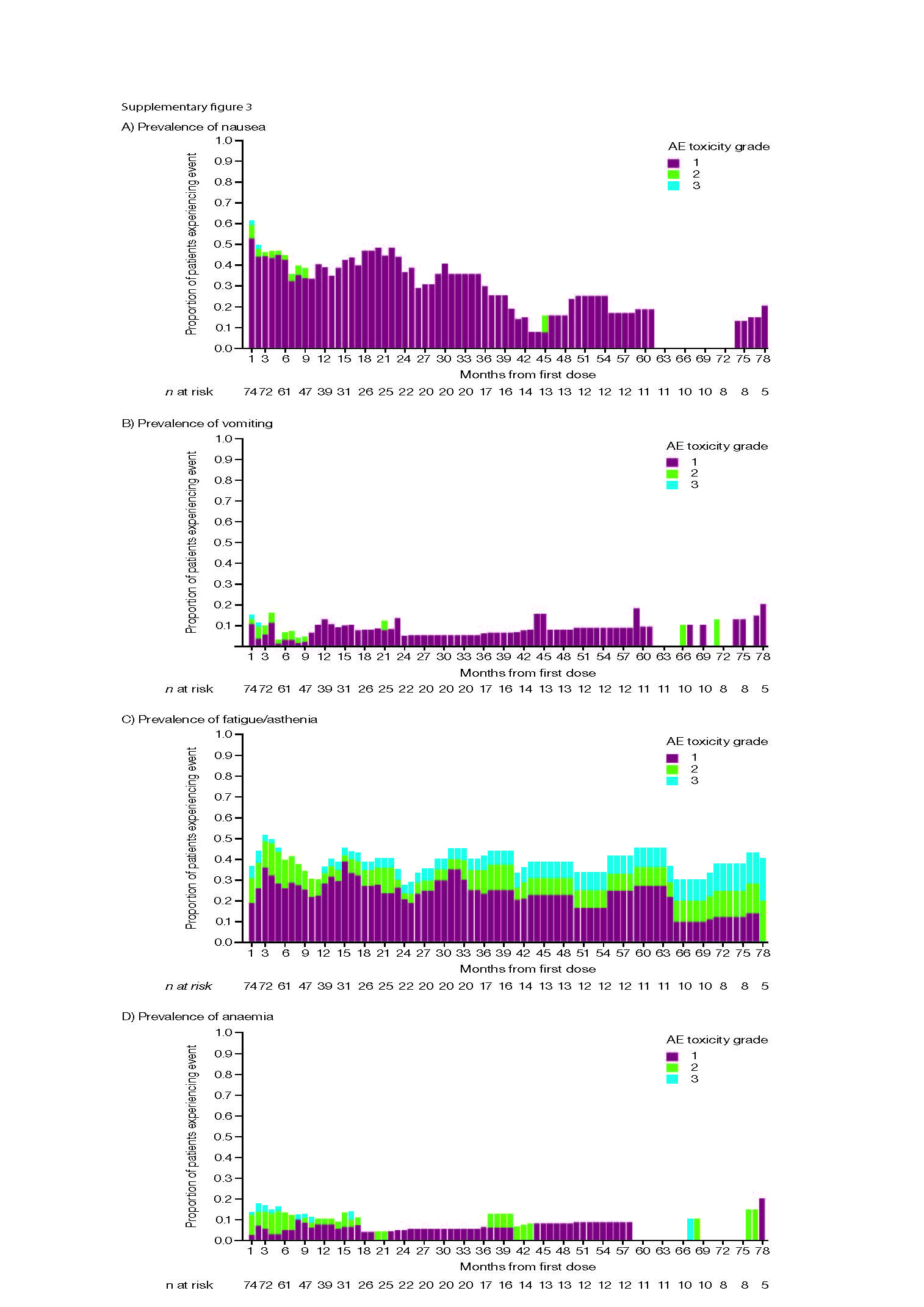


**B** Prevalence of vomiting


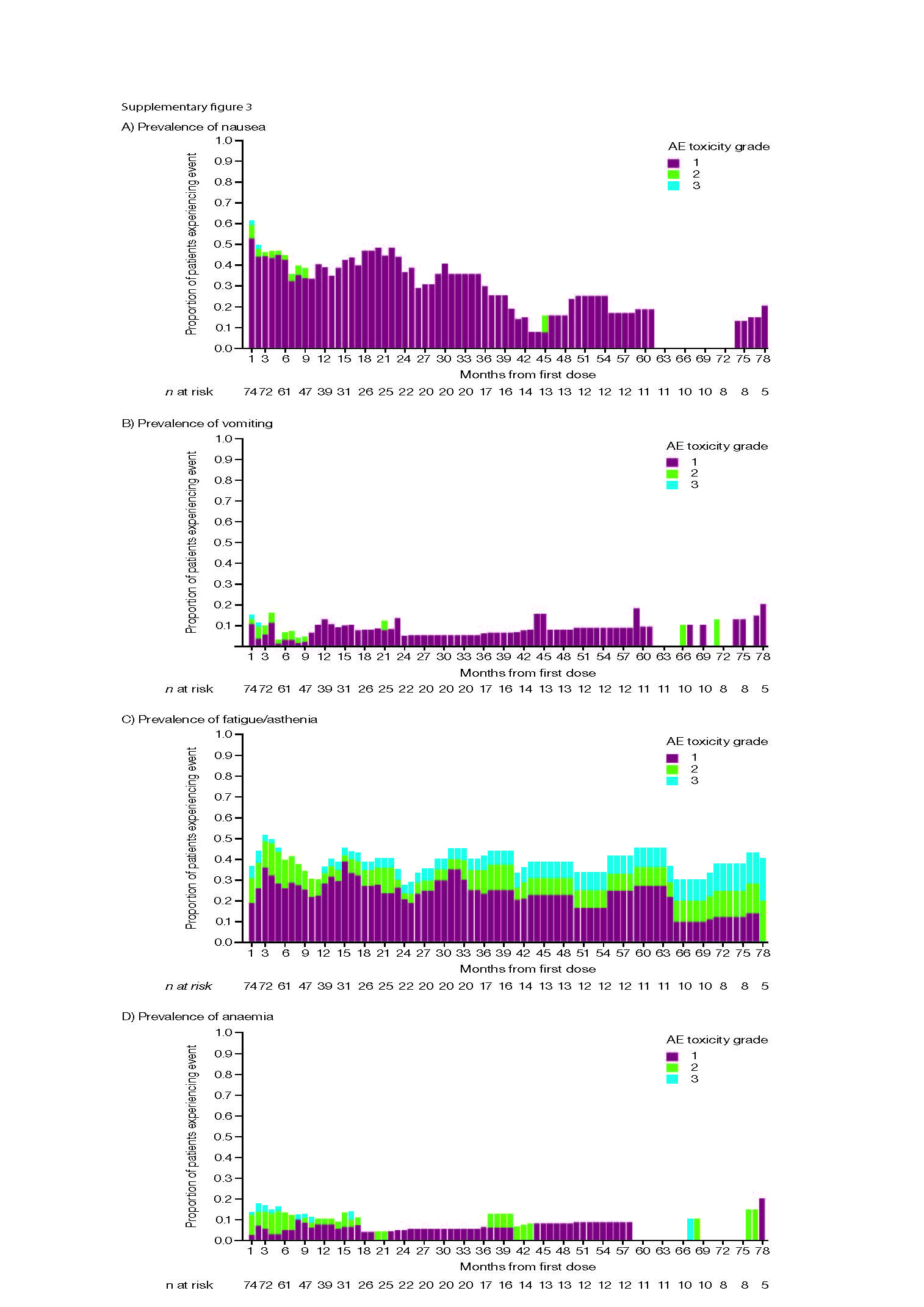


**C** Prevalence of fatigue/asthenia

**
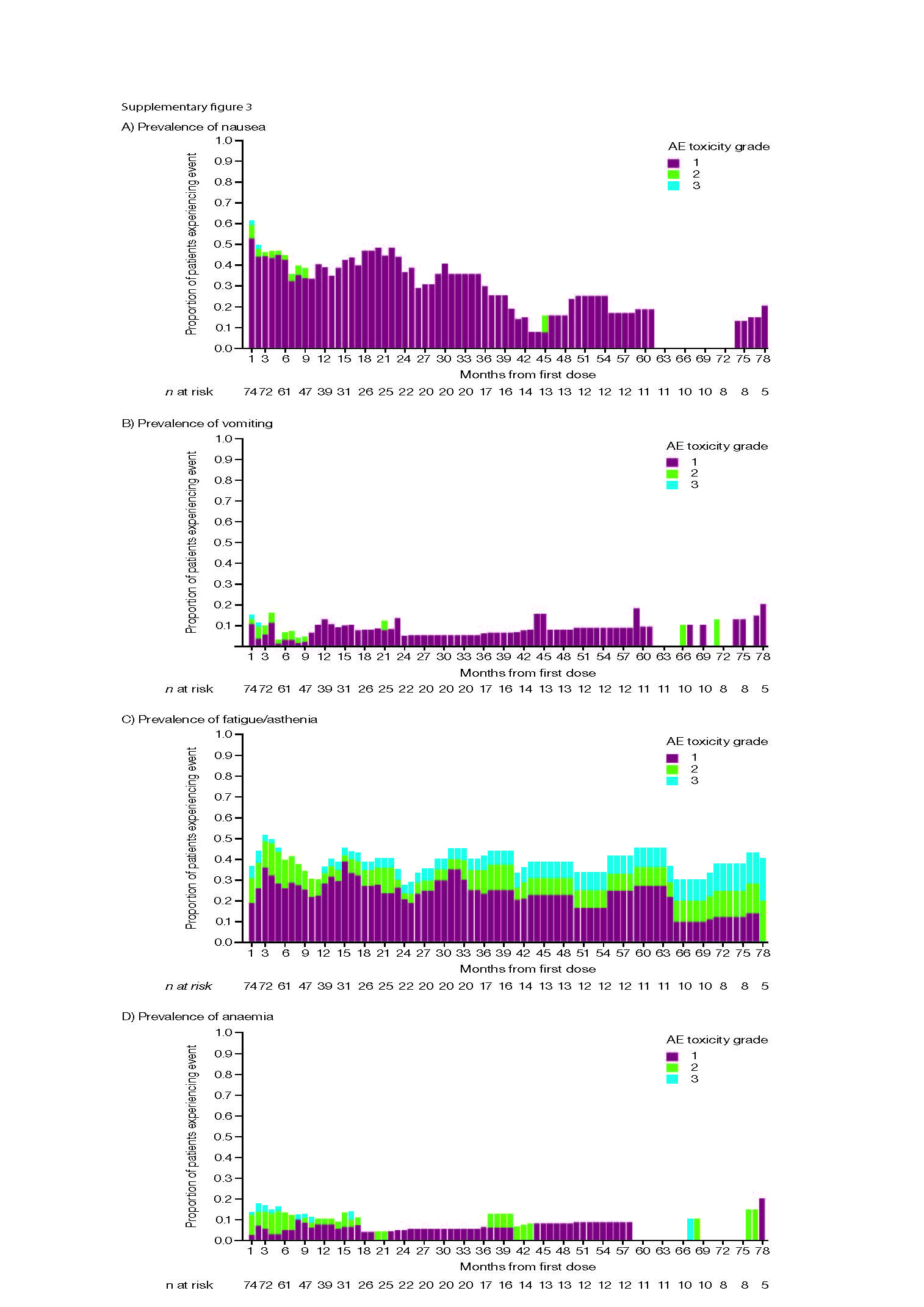
**

**D** Prevalence of anaemia*


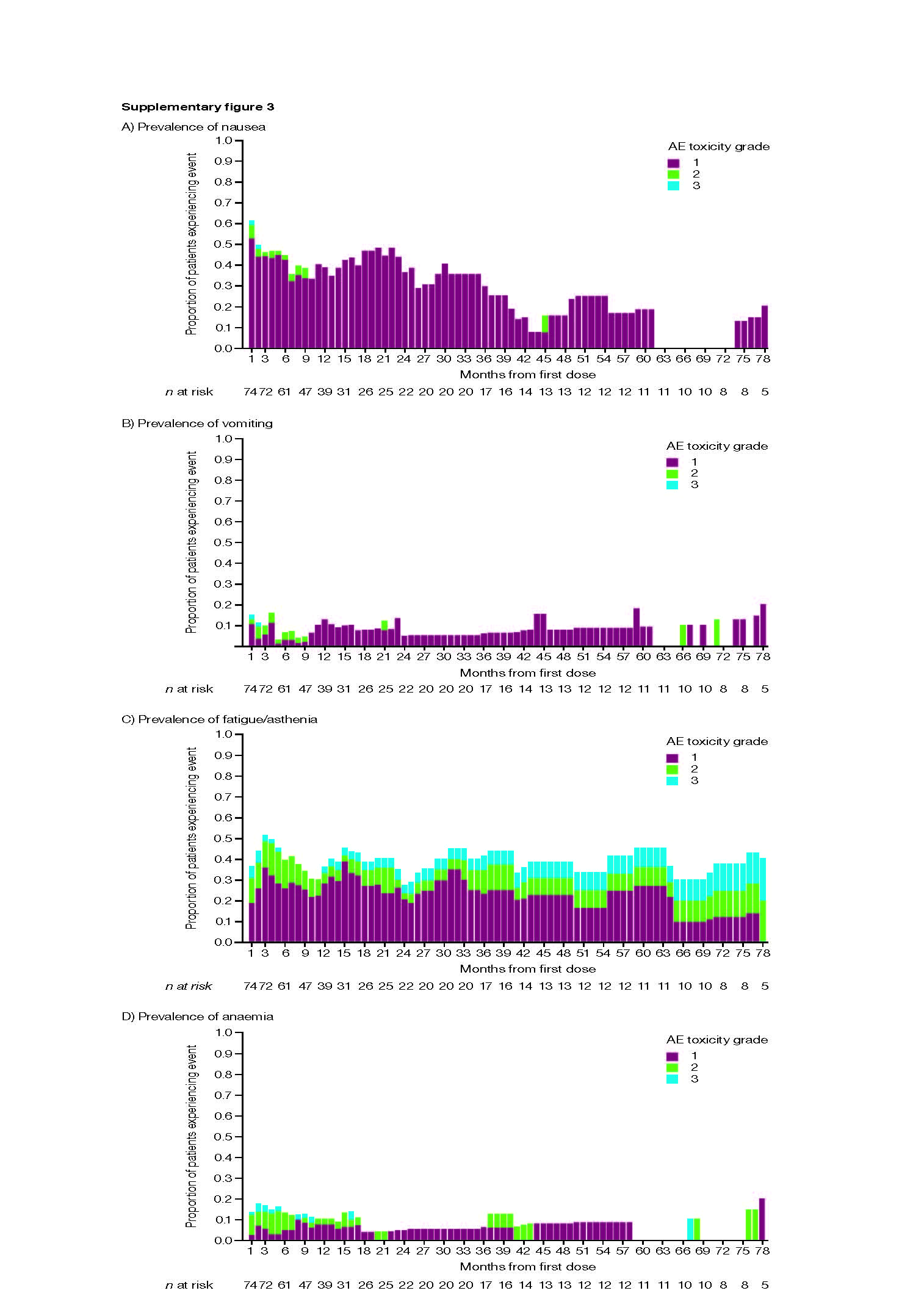


*Includes patients with anaemia, haemoglobin decreased, red blood cell count decreased and haematocrit decreased

**Supplementary references**

1. Myriad Genetics Laboratories I. BRACAnalysis CDx^TM^ Technical Information. 2017. Available at: <https://myriad-library.s3.amazonaws.com/technical-specifications/BRACAnalysis_CDx_Tech_Specs.pdf>.

2. Frampton GM, Fichtenholtz A, Otto GA, Wang K, Downing SR, He J *et al*. Development and validation of a clinical cancer genomic profiling test based on massively parallel DNA sequencing. *Nat Biotechnol* 2013; **31**: 1023-1031.

3. Ledermann J, Harter P, Gourley C, Friedlander M, Vergote I, Rustin G *et al*. Olaparib maintenance therapy in patients with platinum-sensitive relapsed serous ovarian cancer: a preplanned retrospective analysis of outcomes by *BRCA* status in a randomised phase 2 trial. *Lancet Oncol* 2014; **15**: 852-861.

4. Ledermann JA, Harter P, Gourley C, Friedlander M, Vergote I, Rustin G *et al*. Overall survival in patients with platinum-sensitive recurrent serous ovarian cancer receiving olaparib maintenance monotherapy: an updated analysis from a randomised, placebo-controlled, double-blind, phase 2 trial. *Lancet Oncol* 2016; **17**: 1579-1589.

5. Ledermann J, Harter P, Gourley C, Friedlander M, Vergote I, Rustin G *et al*. Olaparib maintenance therapy in platinum-sensitive relapsed ovarian cancer. *N Engl J Med* 2012; **366**: 1382-1392.
